# DeviCNV: detection and visualization of exon-level copy number variants in targeted next-generation sequencing data

**DOI:** 10.1186/s12859-018-2409-6

**Published:** 2018-10-16

**Authors:** Yeeok Kang, Seong-Hyeuk Nam, Kyung Sun Park, Yoonjung Kim, Jong-Won Kim, Eunjung Lee, Jung Min Ko, Kyung-A Lee, Inho Park

**Affiliations:** 1 SD Genomics Co., Ltd., 11F, Seoul Gangnam Post Office, 619 Gaepo-ro, Gangnam-gu, Seoul, 06336 Republic of Korea; 2 Department of Bio and Brain Engineering, KAIST, 291 Daehak-ro, Yuseong-gu, Daejeon, Republic of Korea; 3 Department of Laboratory Medicine, Yonsei University College of Medicine, 211 Eonjuro, Gangnam-gu, Seoul, 06273 Republic of Korea; 4 Department of Laboratory Medicine and Genetics, Samsung Medical Center, Sungkyunkwan University School of Medicine, Seoul, Republic of Korea; 5 Division of Genetics and Genomics, Boston Children’s Hospital and Harvard Medical School, Boston, USA; 6 Department of Pediatrics, Seoul National University Children’s Hospital, Seoul National University College of Medicine, Seoul, Republic of Korea

**Keywords:** Copy-number variation, Targeted sequencing, Visualization, Germ-line, Exon-level

## Abstract

**Background:**

Targeted next-generation sequencing (NGS) is increasingly being adopted in clinical laboratories for genomic diagnostic tests.

**Results:**

We developed a new computational method, DeviCNV, intended for the detection of exon-level copy number variants (CNVs) in targeted NGS data. DeviCNV builds linear regression models with bootstrapping for every probe to capture the relationship between read depth of an individual probe and the median of read depth values of all probes in the sample. From the regression models, it estimates the read depth ratio of the observed and predicted read depth with confidence interval for each probe which is applied to a circular binary segmentation (CBS) algorithm to obtain CNV candidates. Then, it assigns confidence scores to those candidates based on the reliability and strength of the CNV signals inferred from the read depth ratios of the probes within them. Finally, it also provides gene-centric plots with confidence levels of CNV candidates for visual inspection. We applied DeviCNV to targeted NGS data generated for newborn screening and demonstrated its ability to detect novel pathogenic CNVs from clinical samples.

**Conclusions:**

We propose a new pragmatic method for detecting CNVs in targeted NGS data with an intuitive visualization and a systematic method to assign confidence scores for candidate CNVs. Since DeviCNV was developed for use in clinical diagnosis, sensitivity is increased by the detection of exon-level CNVs.

**Electronic supplementary material:**

The online version of this article (10.1186/s12859-018-2409-6) contains supplementary material, which is available to authorized users.

## Background

Targeted next-generation sequencing (NGS) is increasingly being adopted in clinical laboratories for genomic diagnostic tests [1–6]. In addition to single-nucleotide and short insertion/deletion variants (SNVs and INDELs), copy number variants (CNVs) have been implicated as the cause of many human diseases [7, 8] such as HIV [9], rheumatoid arthritis [10], Crohn’s disease [11], psoriasis [12], cancers [13, 14], and inherited rare diseases [15, 16]. However, accurately detecting CNVs in targeted NGS data is challenging because the depth of coverage of targeted NGS data is highly variable over target regions, and regions near breakpoints may not be sequenced [7, 17–22].

For NGS-based CNV detection, there are two major approaches: read-depth and paired-ends mapping methods [1–3, 23–28]. Read-depth based methods detect a CNV by comparing the observed number of mapped reads with the expected number of mapped reads in a genomic interval [29]. The calculation of the expected number of mapped reads in a genomic interval assumes a neutral copy number in that interval. Paired-ends mapping based methods identify a CNV by looking for concordantly mapped paired-ends reads whose insert sizes are deviated significantly from the distribution of insert sizes in a sequencing library [19].

In general, paired-ends based methods can predict CNV breakpoints more precisely [19], but it is difficult to apply these methods to targeted NGS data because genomic regions near breakpoints are difficult to sequence. Read-depth based methods are more frequently applied to targeted NGS data because they are less affected by the above limitation. However, currently available read-depth based methods suffer from high false positive predictions, especially on detection of small CNVs spanning only one or a few exons, which may be a hurdle for the adoption of these methods in clinical diagnosis [4]. Because small CNVs have been casually implicated in many inherited disorders [30], accurate detection of small CNVs is important in improving the diagnostic performance of targeted NGS based clinical tests.

For the clinical use of targeted NGS, visual inspection of the detected variants in the regions of genes suspected to be responsible for the disease of a given patient is a crucial step before clinical interpretation [1]. Visual inspection allows for selection of variants that are worth further validation with orthogonal methods such as qPCR, and lowers the risk of missing true pathogenic variants such as CNVs that might be difficult to detect with conventional methods. The latter is especially important for genes that are clinically relevant to the phenotype of a given patient or that have a pathogenic heterozygous sequence variant in recessive Mendelian disorders.

Here, we developed a new method, DeviCNV, to meet the two clinical requirements for CNV detection using targeted NGS data: 1) the detection of CNVs with exon-level resolution, and 2) the support of intuitive visualization for the assessment of CNVs. To meet the first requirement, we attempted to fully exploit detailed CNV signals from target capture probes for gene panels. Probe level data, which even a single exon can have multiple, allow DeviCNV to assign confidence scores to the CNV candidates based on the reliability and strength of the CNV signals calculated from the multiple probes. It also provides gene-centric view plots with confidence levels of the CNV candidates of a gene. The gene-centric view plots show the read-depth ratios of the probes within the gene with their confidence intervals and the probabilities of their read depth ratios being outside the ranges of copy neutral.

## Results

### Dataset and parameter setting

We sequenced 27 cell lines with inherited genetic disorders obtained from the NHGRI Sample Repository for Human Genetic Research at the Coriell Institute for Medical Research as targeted NGS data: lymphoblastoid cell lines/DNA samples from adrenal hyperplasia patients (NA11781, NA12217, NA14734, GM14734), a galactosemia patient (GM17433), a type I gaucher disease patient (NA10874), glycogen storage disease II patients (GM14011, GM14259, GM14603), a krabbe disease patient (NA06805), lesch-nyhan syndrome patients (NA01899, NA06804), transcarbamylase deficiency patients (GM23431, GM23891, GM24007), phenylketonuria patients (NA02659, NA11195), propionic academia patients (NA22208, NA22496, NA22555, GM23221) and as a control sample (NA12878), and fibroblasts cell lines/DNA samples from a galactosemia patient (NA01741), a type I gaucher disease patient (NA00852), a lesch-nyhan syndrome patient (NA02227) and phenylketonuria patients (NA00006, NA02406). Eight of them are known to have pathogenic CNVs. We used those pathogenic CNVs as a standard answer set for parameter optimization of DeviCNV. These 27 cell lines were sequenced using target gene panels IMD_HYB, IMD_PCR, or both (Table 1). Both IMD_HYB and IMD_PCR are target gene panels for NGS designed for identifying genetic variants responsible for newborn screening disorders. IMD_HYB and IMD_PCR are developed with hybridization-based and PCR-based target enrichment technologies respectively. All the sequencing data for these cell lines were submitted to the NCBI Short Read Archive databank (SRA, http://www.ncbi.nlm.nih.gov/sra) under accession number SRP103698 (SRA).

**Table 1:** Summary of the dataset used for retrospective and clinical analyses

Gene panel name	Capture method	Number of target genes	Probes (or amplicons)	Probe coverage size	Average number of probes per exon	Clinical use	Number of samples
IMD_HYB	Hybridization (HiSeq)	259	19210	982,657 bps	5.7	Newborn screening	30^a^ (cell line) 36 (clinical)
IMD_PCR	PCR (Ion S5)	259	9072 (3 pools)	1,216,913 bps	2.7	Newborn screening	14 (cell line) 20 (clinical)
IMD_V1	PCR (Ion PGM)	97	2054 (2 pools)	338,961 bps	1.8	Newborn screening	178 (clinical)

*IMD* inherited metabolism disorder, *HYB* hybridization-based capture approach, *PCR* polymerase chain reaction-based capture approach, *bps* base pairs

^a^27 unique cell line. Total 30 samples were sequenced because two cell lines were generated 3 times respectively

The average of mean target depths for these cell lines were 174X for the IMD_HYB dataset and 301X for the IMD_PCR dataset (Table 2). As for the minimum of mean target depth of a sample eligible for CNV detection, we recommend 100X for the IMD_HYB dataset and 150X for the IMD_PCR dataset (Additional file 1: Note S1). Another aspect of the quality of targeted NGS data of a sample is measured by coefficients of correlation of read depth values of probes with the other samples within the same sequencing batch (described in the Method section). We excluded a sample in CNV detection if the sample has low coefficients of correlation with the other samples.

**Table 2:** Summary of cell lines and clinical cohorts

Panels	IMD_HYB		IMD_PCR		IMD_V1
Batches	3		2		Unknown
Samples	30^a^ (cell line)	36 (clinical)	14 (cell line)	20 (clinical)	178 (clinical)
Average depth of coverage	174X	345X	301X	349X	87X
Samples passing QC	24	35	14	19	172
Failure rate	20%	2.8%	0%	5%	3.4%
Median number of raw duplications	52.5	8	35.5	29	22.5
Median number of raw deletions	22.5	3	37	23	9
Median number of raw CNVs	82	13	85.5	67	34.5
Median number of 5-score^b^ duplications	4.5	1	12	5	6
Median number of 5-score deletions	2	0	5.5	2	1
Median number of 5-score CNVs	6.5	1	24.5	7	7.5

*QC* quality control, *CNV* copy number variation, *IMD* inherited metabolism disorder, *HYB* hybridization-based capture approach, *PCR* polymerase chain reaction-based capture approach

^a^27 unique cell line. Total 30 samples were sequenced because two cell lines were generated 3 times respectively

^b^High-confidence CNVs received the highest score of 5

Because DeviCNV aims to detect exon level CNVs with high sensitivity, it keeps every CNV candidates by categorizing with their confidence score rather than hard filtering of low confidence CNV candidates. To measure the confidence score, we introduce the five criteria which reflect the reliability and strength of CNV signals of the candidates (Table 3): 1) *ProbeCntInRegion*, 2) *AverageOfReadDepthRatios*, 3) *STDOfReadDepthRatios*, 4) *AverageOfCIs*, and 5) *AverageOfR2vals*. These criteria consider the number of probes, the strength of CNV signals, the stability of read depth ratios, and reliability of regression models among the probes within a CNV candidate region.

**Table 3:** Description of the measures used in the DeviCNV scoring system

Abbreviation	Description	Calculation method	Default parameter setting
ProbeCntInRegion	How many signals support the CNV candidate?	Counting read depth ratio signals for a CNV candidate	1 point for ≥2
AverageOfReadDepthRatios	How strong is the signal supporting the CNV candidate?	Calculating an average log2-transformed median predicted probe-level read depth ratio values for a CNV candidate	If deletion, 1 point for < log2(0.6); If duplication, 1 point for > log2(1.4)
STDOfReadDepthRatios	How stable are the signals supporting the CNV candidate?	Calculating a standard deviation for the log2-transformed median predicted probe-level read depth ratio values for a CNV candidate	1 point for < 0.4
AverageOfCIs	How small are the confidence intervals for the signals supporting the CNV candidate?	Calculating average log2-transformed 95% confidence interval lengths for predicted probe-level read-depth ratios for a CNV candidate	1 point for < 0.4
AverageOfR2vals	How reliable is the model that generated the signals that support the CNV candidate?	Calculating average mean R-squared values per probe for a CNV candidate, with the average R-squared value per probe referring to an average of the R-squared values of N models for one probe	1 point for ≥0.85

*CNV* copy number variant, *CI* confidence interval

DeviCNV counts how many of the above criteria are satisfied for each CNV candidate. For each criterion, we selected the thresholds or conditions by minimizing the number of CNV candidates satisfying the criterion, while all the known pathogenic CNVs are preserved. We excluded deletions in *CYP21A2* because the deletions in the gene is known to be challenging to detect with NGS data due to its pseudogene and copy number polymorphisms [31]. The default thresholds and conditions for those criteria are shown in Table 3. If a CNV candidate satisfies all the above five criteria, it scores 5. The CNV candidates with the highest score are considered as the top priority for visual inspection.

### Concordance with qPCR of CNV candidates detected from DeviCNV

To evaluate the performance of DeviCNV, we performed qPCR on the subset of CNV candidates with confidence score of 5 from the IMD_HYB dataset. The subset was selected from 11 cell lines with the number of CNV candidates of score 5 less than 10, which resulted in a total of 40 CNV candidates (27 duplications and 13 deletions). Apart from four already known pathogenic CNVs, 36 CNV candidates were tested by qPCR (Additional file 1: Note S2), and 11 out of the 27 duplications, and five out of the nine deletions were confirmed by qPCR. In addition, we randomly selected 25 of the 497 CNV candidates with confidence score of 4 from the above 11 cell lines. Of these 25 CNVs, 6 out of the 16 duplicates and 3 out of the 9 deletions were also confirmed by qPCR (Additional file 1: Note S2). As a summary, the concordance rates of 5-score CNV candidates and 4-score CNV candidates were 44% (16 out of 36) and 36% (9 out of 25) respectively.

### Comparison with other tools

We compared DeviCNV’s germline exon-level CNV detection performance with VisCap [1], XHMM [2], and CODEX [27] using the IMD_HYB dataset and the IMD_PCR dataset.

From the IMD_HYB dataset and the IMD_PCR dataset, DeviCNV, VisCap, XHMM, and CODEX could each detect 11, eight, eight, and eight out of 14 known CNVs (eight known CNVs from the IMD_HYB dataset and six known CNVs from the IMD_PCR dataset) respectively (Table 4). Notably, DeviCNV is the only tool which found all the small CNVs spanning over four or less exons: the deletion of exon 18 of *GAA* from GM14603, and the duplication of exon 2 and 3 of *HPRT1* from NA06804. As for the total number of CNV candidates, DeviCNV was comparable with a median of 9.5 CNV candidates per sample. The other tools VisCap, XHMM, and CODEX generate a median of 15.5, 2.0, and 26.0 CNV candidates per sample, respectively.

**Table 4:** Comparison of the performances of DeviCNV and previous tools using cell lines with known CNVs

Sample			Known CNV				DeviCNV		VisCap		XHMM		CODEX	
Panel	Cell line	Median read depth	Gene	NM	CNV	CNV size (kb)	Find?^a^	#CNV^b^	Find?	#CNV	Find?	#CNV	Find?	#CNV
IMD_HYB	GM14603	81.99	*GAA*	NM_000152	EX18 DEL	0.16	O	24	X	7	X	0	X	56
	GM14734	249.4	*CYP21A2*	NM_000500	30 KB DEL, Entire gene DEL	3.35	O	2	O	37	O	1	X	2
	GM24007	142.84	*OTC*	NM_000531	Entire gene DEL	68.97	O	7	O	14	O	3	O	46
	NA01741	164.4	*GALT*	NM_000155	Entire gene DEL	4.01	O	6	O	40	O	1	O	37
	NA06804	261.98	*HPRT1*	NM_000194	EX2–3 DUP	2.01	O	34	O	43	X	2	O	62
	NA06805	80.13	*GALC*	NM_000153	EX11–17 DEL	17.73	O	44	O	8	O	1	O	86
	NA12217	269.08	*CYP21A2*	NM_000500	30 KB DEL	1.14	X	1	X	7	X	3	X	11
	NA22208	199.64	*PCCA*	NM_000282	EX13–20 DEL	146.38	O	3	O	17	O	2	O	15
IMD_PCR	NA01741	Pool 1: 408.0, Pool 2: 556.0, Pool 3: 271.0	*GALT*	NM_000155	Entire gene DEL	4.01	O	10	O	9	X	0	O	1
	NA12217	Pool 1: 192.0, Pool 2: 117.0, Pool 3: 99.0	*CYP21A2*	NM_000500	30 KB DEL	1.14	X	37	X	22	X	8	X	71
	GM14603	Pool 1: 215.0, Pool 2: 141.0, Pool 3: 90.0	*GAA*	NM_000152	EX18 DEL	0.16	O	25	X	32	O	6	O	40
	NA14734	Pool 1: 359.0, Pool 2: 275.0, Pool 3: 335.0	*CYP21A2*	NM_000500	30 KB DEL, Entire gene DEL	3.35	O	9	O	12	O	4	X	12
	NA22208	Pool 1: 235.0, Pool 2: 99.0, Pool 3: 158.0	*PCCA*	NM_000282	EX13–20 DEL	146.38	O	27	X	13	O	4	O	12
	GM24007	Pool 1: 37.0, Pool 2: 20.0, Pool 3: 16.0	*OTC*	NM_000531	Entire gene DEL	68.97	X	1	X	23	X	0	X	0

*CNV* copy number variation, *IMD* inherited metabolism disorder; *HYB* hybridization-based capture approach, *PCR* polymerase chain reaction-based capture approach, *EX* exon, *DEL* deletion, *DUP* duplication

^a^Indicates whether a known CNV was found using each tool. “O” means all CNVs were found, and “X” means they were not found at all

^b^indicates the number of CNV candidates found in the corresponding sample. For DeviCNV, the number of CNV candidates that received the highest score of 5 is indicated

We also evaluated how many of the 5-score CNVs confirmed by qPCR could be detected with other methods. Among 16 CNVs validated with qPCR, VisCap, XHMM, and CODEX could detect two, two, and five CNVs, respectively. (Table 5 and Additional file 1: Note S3). Most of those 16 CNVs are consists of one or two exons implying DeviCNV can detect CNVs that only span over a length of one or two exons which the other tools did not detect well.

**Table 5:** Comparison of the performances of DeviCNV and previous tools using 16 CNVs confirmed by qPCR

Sample		qPCR confirmed CNV				DeviCNV	VisCap	XHMM	CODEX
Sample	Median read depth	Gene	NM	CNV	CNV size (kb)				
GM17433	82.13	*CPT1A*	NM_001876	EX10 DUP	0.20	O^a^	X	X	X
		*CD3E*	NM_000733	EX4 DEL	0.01	O	X	X	O
		*GATM*	NM_001482	EX9 DUP	1.10	O	X	X	X
GM24007	142.84	*PTPRC*	NM_002838	EX16–17	0.83	O	X	X	O
		*LMBRD1*	NM_018368	EX12 DUP	0.10	O	X	X	X
		*SLCO1B3*	NM_019844	EX4 DUP	0.14	O	X	X	O
		*PAH*	NM_000277	EX5 DEL	0.07	O	O	X	X
		*NR0B1*	NM_000475	EX1 DEL	1.18	O	O	X	O
NA00852	204.09	*HBA2*	NM_000517	EX2–3 DEL	0.59	O	X	O	X
NA01741	164.4	*TG*	NM_003235	EX20 DUP	0.22	O	X	X	X
		*TG*	NM_003235	EX 21 DUP	0.15	O	X	X	X
NA02227	278.98	*CYP21A2*	NM_000500	EX10 DUP	0.80	O	X	X	X
NA02659	608.46	*HBA2*	NM_000517	EX3 DEL	0.24	O	X	O	X
NA12217	269.08	*GBA*	NM_001005741	EX12–11 DUP	0.86	O	X	X	X
NA22496	137.24	*GUSB*	NM_000181	EX11 DUP	0.14	O	X	X	X
		*G6PC*	NM_000151	EX2 DUP	0.11	O	X	X	O

*CNV* copy number variation, *EX* exon, *DEL* deletion, *DUP* duplication

^a^Indicates whether a known CNV was found using each tool. “O” means all CNVs were found, and “X” means they were not found at all

#### Identification of pathogenic CNVs associated with inherited metabolic disorders

We used DeviCNV to detect CNVs in clinical samples suspected of having inherited metabolic disorders. We collected clinical samples from three cohorts (Table 2 and Additional file 1: Note S4).

**Fig. 1:**
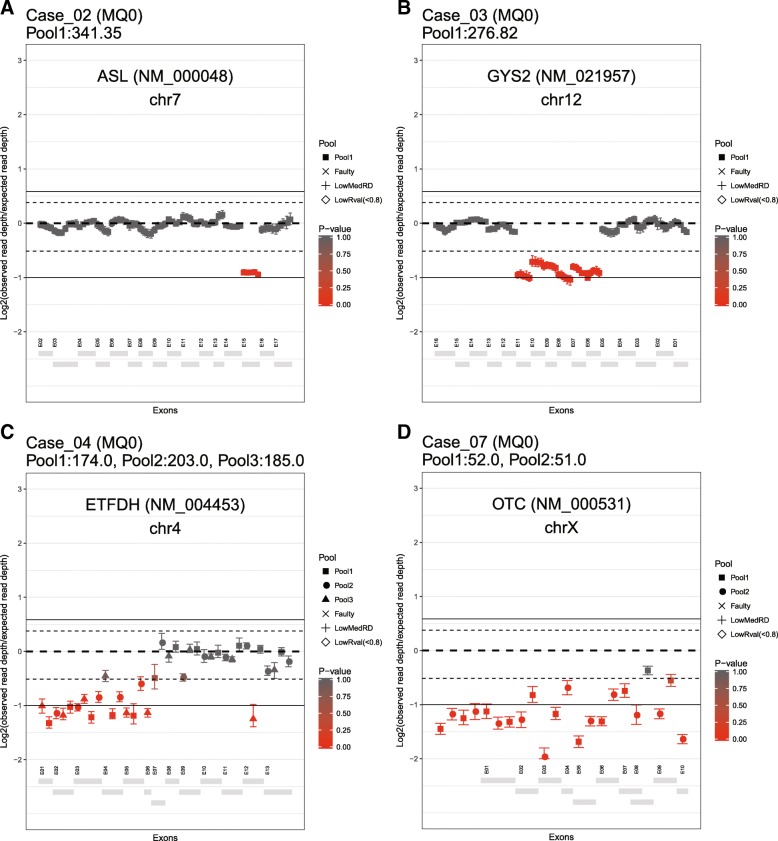
Gene-centric view plots for four selected clinical cases. Panels A–D contain four examples of gene-centric view plots for the pathogenic CNVs detected in clinical samples shown in Table 6. **a** A single exon deletion within *ASL*, **b** a multi-exon deletion within *GYS2* using the inherited metabolic disorder panel and hybridization capture approach, **c** a multi-exon deletion within *ETFDH* using the inherited metabolic disorder panel and polymerase chain reaction-based capture approach, and **d** an entire gene deletion within *OTC* using the previous version of the inherited metabolic disorder panel and polymerase chain reaction-based capture approach

In total, we sequenced 45 clinical samples using either IMD_HYB or IMD_PCR or both. Of these 45 samples, 36 samples were sequenced with IMD_HYB with an average of mean target depths of 345X, while 20 samples were sequenced with IMD_PCR with an average of mean target depths of 349X. From the results of DeviCNV, our clinical reviewers selected the five CNV candidates for further validation by integrating the sequence variants (SNVs and INDELs) and clinical information of patients (Additional file 1: Note S5). Among the five selected CNV candidates, four CNVs were confirmed by qPCR (Table 6 and Fig. 1).

**Table 6:** Candidate pathogenic CNVs detected by clinical sample analysis using DeviCNV

Sample			CNV candidates after scoring^a^							Selected pathogenic CNVs^c^				
Panel	Sample	Median read depth	Raw CNV^b^	Score 5	Score 4	Score 3	Score 2	Score 1	Score 0	Gene	NM	CNV	CNV size (kb)	Confirmed by qPCR
IMD_HYB	Case_01	273.3	49	2	22	20	5	0	0	*ACADVL*	NM_000018	EX2 DEL (Score 4)	0.08	Failed
	Case_02	341.4	12	7	3	2	0	0	0	*ASL*	NM_000048	EX15 DEL (Score 5)	0.08	Confirmed
	Case_03	276.8	25	5	18	2	0	0	0	*GYS2*	NM_021957	EX6–11 DEL (Score 5)	5.15	Partially confirmed (EX6–7, 10–11)
IMD_PCR	Case_04	Pool 1: 174.0 Pool 2: 203.0 Pool 3: 185.0	82	26	46	9	1	0	0	*ETFDH*	NM_004453	EX1–7 DEL (Score 5)	23.51	Confirmed
	Case_05	Pool 1: 228.0 Pool 2: 330.0 Pool 3: 185.0	145	63	74	8	0	0	0	*ETFDH*	NM_004453	EX7–8 DEL (Score 5)	2.20	Confirmed
IMD_V1	Case_06	Pool 1: 69.0 Pool 2: 56.0	106	37	40	26	3	0	0	*OTC*	NM_000531	EX2 DEL (Score 5)	0.14	Confirmed
	Case_07	Pool 1: 52.0 Pool 2: 51.0	65	23	23	14	5	0	0	*OTC*	NM_000531	Entire gene DEL (Score 5)	68.38	Confirmed

*CNV* copy number variation, *IMD* inherited metabolism disorder; *HYB* hybridization-based capture approach, *PCR* polymerase chain reaction-based capture approach, *EX* exon, *DEL* deletion, *DUP* duplication, *qPCR* quantitative polymerase chain reaction

^a^Indicates the number of CNV candidates for each score

^b^indicates the number of all CNV candidates before scoring

^c^indicates the selected pathogenic CNVs identified in the clinical sample by one expert. The number in parentheses indicates the score of the selected CNV

We also analyzed 178 samples sequenced using IMD_V1, previous version of IMD_PCR (Table 2), which had an average of mean target depths of 87X. We ran DeviCNV on 172 samples that passed the quality control, as an input set because lacking sequencing batch information. Our clinical reviewers chose two CNVs for further validation, and these were all confirmed by qPCR.

## Discussion

DeviCNV was optimized with the known pathogenic CNVs whose parameters are set to detect all the known CNVs except for deletions of *CYP21A2*. It was further evaluated by qPCR for the high confidence CNV candidates generated with DeviCNV. We observed that the quality of sequencing of samples are critical to reduce the number of CNV candidates while retaining the true CNVs. Thus, we suggest the minimum requirement of the input samples for the proper use of DeviCNV. We also used DeviCNV on clinical samples, and successfully identified six disease-associated CNVs (Table 6) that leads to conclusive clinical diagnosis.

## Conclusion

Although targeted NGS is becoming a major diagnostic and screening method to detect genomic variants, it still is challenging to detect CNVs in targeted NGS data with confidence. Here, we propose a new pragmatic method for detecting CNVs in targeted NGS data that includes visualization functionality and confidence scores for clinical interpretation. Since DeviCNV was developed with the intention of use in clinical diagnosis, sensitivity was emphasized for the detection of exon-level CNVs. We developed two submodules of DeviCNV to be used with two popular targeted NGS approaches: hybridization- and PCR-based capture approaches. DeviCNV provides visualization plots that support the clinical interpretation of the clinical reviewer by offering confidence levels that reflect the quality of the sequencing data of a sample, the reliability of the regression models for probes and their read depth ratios. By integrating sequence variants and novel CNVs detected by DeviCNV, our clinical reviewers could make conclusive diagnosis for several patients.

## Methods

### Overview of DeviCNV

DeviCNV can be divided into three main components: 1) calculation of the probe (or amplicon)-level ratio of the observed and estimated read depth based on linear regression models of the read depth of a probe and the median read depth values of all probes in a sample, 2) generation of CNV candidates by applying a circular binary segmentation (CBS) algorithm to the read depth ratios of probes, and assigning confidence scores for them with the five scoring criteria based on the probe-level CNV signals within candidates, and 3) visualization of the CNV candidates with confidence information for easier visual inspection.

**Fig. 2:**
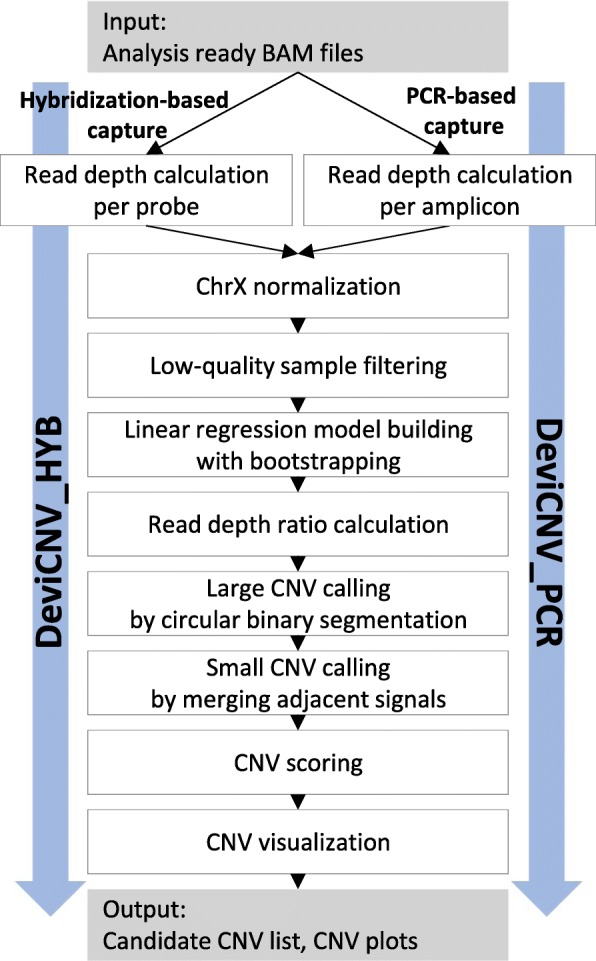
DeviCNV workflow. Analysis-ready BAM files were used for DeviCNV input. After read-depth normalization for chromosome X, DeviCNV filters low-quality samples from the input dataset. Then, DeviCNV builds N (1,000 by default) linear regressions per probe (or amplicon) to predict a read-depth ratio and confidence interval per probe for each sample. By combining signals of probe-level read-depth ratios, DeviCNV calls raw CNV candidates and evaluates them using a new scoring system. Finally, DeviCNV provides a CNV candidate list and visualization plots for each sample and gene

To calculate the probe-level read depth ratios, we implemented two submodules to be used in two popular NGS target enrichment approaches: hybridization- and polymerase chain reaction (PCR)-based capture approaches (Fig. 2). Hereafter, we use the terms “probe” and “amplicon” interchangeably without the loss of generality with respect to the calculation of read depth ratio for a target capture interval.

### Input for DeviCNV

DeviCNV requires three inputs: 1) binary alignment/map (BAM) formatted files for a set of samples, 2) a tab delimited text file that contains the genomic position of target capture probes or amplicons with their primer/probe pool information, and 3) the genders of the samples. Because DeviCNV uses linear regression models to estimate probe-level read depth ratios, a minimum number (≥ 6) of samples is recommended to build the models properly (Additional file 1: Note S6). Using BAM files of samples from a batch of sequencing run is also recommended to rule out batch effects (Additional file 1: Note S7).

### Calculating probe level read depth

Many previous studies have used individual exons or unified regions merged with overlapping probes as units for calculating read depth. However, these approaches overlook the usefulness of the detailed probe-level signals which may be helpful in determining the confidence of CNV candidates [23]. Our premise of using probe-level signals for calling CNVs is that if there are CNV signals from multiple probes for a candidate, then we could give more confidence to the candidate even in a single exon sized CNV. Therefore, DeviCNV uses each individual probe as units to detect CNV signals, rather than individual exons or unified regions as units (Additional file 1: Note S8).

To calculate probe-level read depth, DeviCNV counts the number of sequencing reads mapped to a probe region with a mapping quality value (MQV) threshold. However, we observed that there is no recognizable difference in terms of performance between the default MQV ≥ 0 and the MQV ≥ 20 (Additional file 1: Note S9).

The two submodules for calculating probe-level read depth are described as followed:

- *PCR based capture-specific approach*. Most sequencing reads can be assigned to an amplicon from which sequencing reads were generated from. For a given sequencing read, DeviCNV selects the amplicon that overlaps most with the aligned genomic interval. If two or more amplicons have the same overlap ratio for the sequencing read, the smallest amplicon among them is assigned.
- *Hybridization based capture-specific approach*. In hybridization based targeted NGS, sequencing reads captured by a target capture probe originated from many physically different molecules, resulting in different alignment for those sequencing reads. Therefore, it is not trivial to determine which target capture probe was a bait for a sequencing read. For this reason, DeviCNV uses the average of per-base depth of coverage within a target capture probe region as the reads depth for that target capture probe.

### X chromosome normalization

To adjust for the different number of X chromosomes in males and females, DeviCNV normalizes the probe-level read depth on the X chromosome by dividing by two in case of females.

### Low-quality sample filtering

In addition to the mean target depth as a quality control for a sample, we calculated coefficients of correlation of its probe level reads depth with those of other samples. To determine the threshold for low quality samples, we investigated the relationship between the coefficients of correlations of a sample with the other samples and the number of segments generated during the CBS with the read depth ratios for the sample (Additional file 1: Note S10). Finally, we excluded a sample for CNV calling if its top quadrant of coefficients of correlations are below 0.7.

### Building linear regression models with bootstrapping

In principal, DeviCNV uses a linear regression model to predict an expected read depth of a probe of a sample with the median of read depth values of all probes in the sample as a predictive variable. To generate empirical distribution of expected read depth of a probe in each sample, DeviCNV builds N linear regression models with N resampling with replacement. Then, it calculates N read depth ratios between the observed read depth and the N expected read depths. Our rationale for using linear regression models is that the read depth of a probe for a given sample should be proportional to a representative quantity of sequencing depth for the sample, if its copy number is neutral. By default, the number of resampling N is set to 1000. The 95% confidence interval of the expected read depth is obtained from this process.

During the building process of N linear regression models, DeviCNV identifies low-quality probes that cannot be used in calling CNV deletion which are categorized into faulty probes, faulty sample of the probe, and low R-squared value probe.

#### Faulty probe

Negative value among the slopes of regression models for a probe during the bootstrapping indicates read depth of the probe does not follow the assumption of proportional relationship between read depth values of the probe and sequencing depths of samples. The results from faulty probes are not considered when calling CNVs across all samples.

#### Faulty sample of the probe

Negative value among the expected read depth values of a probe in a sample during the bootstrapping indicates that the median of read depth values of all probes in a sample is too low to calculate the read depth ratio reliably in the regression models of the probe. Thus, for a given sample, the results from those probes are not considered for CNV calling.

#### Low R-squared value probe

Average R-squared value of the N regression models of a probe under 0.8, indicates the computed linear regression models are not reliable enough to be used in CNV calling. These results are not considered for CNV calling across all samples.

### Calculating read depth ratio per target capture probe

For a given target capture probe t, let *Y*_*t*_ = (*y*_t, 1_, *y*_t, 2_, …, , *y*_t, K_) be the read depth of the probe t observed from the targeted NGS data of the K samples. Median of read depth values of all probes in each sample is denoted as M = (*m*_1_, *m*_2_, …, *m*_K_). Then, we build N linear regression models between M (independent variable) and *Y*_*t*_ (response variable) by resampling with replacement. We denote the N fitted linear regression models of the probe t as *F*_*t*_ = (*f*_t, 1_, *f*_t, 2_, …, *f*_t, N_). From each fitted linear regression model, we can estimate the read depth of a probe t at sample k by the *n*^th^ model with the equation ${\overset{\sim }{y}}_{t,k,n}={f}_{\mathrm{t},\mathrm{n}}\left({m}_{\mathrm{k}}\right)$. Then, we calculate the read depth ratio of the observed read depth and the estimated read depth by ${r}_{t,k,n}=\frac{y_{t,k}}{{\overset{\sim }{y}}_{t,k,n}}$. Finally, we can get N of read depth ratio estimates which we denote as *R*_*t*, *k*_ = (*r*_*t*, *k*, 1_, …, *r*_*t*, *k*, N_) .

To measure the significance of CNV signal from *R*_*t*, *k*_, probability of a CNV event is calculated from the fraction of how many read-depth ratios among its N read depth ratios are deviated from the range of copy neutral defined as (TH.del, TH.dup) where TH.del and TH.dup are the thresholds for deletion and duplication, respectively. The default value is 0.7 for TH.del and 1.3 for TH.dup (Additional file 1: Note S11). Finally, we selected the probes whose probability of a CNV event is greater than 0.5.

$$ p.{dup}_{\left(t,k\right)}=\frac{\mathbf{n}\left({r}_{t,\mathrm{k},n}>\mathrm{TH}.\mathrm{dup}\right)}{\mathrm{N}} $$

$$ \mathrm{If}\ p.{dup}_{\left(t,k\right)}>0.5,\mathrm{then}\ {C}_{\left(t,k\right)}=\mathrm{duplication} $$

$$ p.{del}_{\left(t,k\right)}=\frac{\mathbf{n}\left({r}_{t,k,n}<\mathrm{TH}.\mathrm{del}\right)}{\mathrm{N}} $$

$$ \mathrm{If}\ p.{del}_{\left(t,k\right)}>0.5,\mathrm{then}\ {C}_{\left(t,k\right)}=\mathrm{deletion} $$

(Otherwise,) *C*_(*t*, *k*)_ = neutral

where *C*_(*t*, *k*)_ is the copy number status (duplication/neutral/deletion) for sample k with target capture probe t.

### Calling CNVs

To segment a profile of sample’s read depth ratios for a gene, we used a circular binary segmentation (CBS) method [32]. The profile used in CBS was generated with the medians of *R*_*t*, *k*_ of the probes within a gene. For computational convenience, we set the upper limit of the read depth ratios of the profile as 16.

$$ {P}_{\left(t,k\right)}=\mathrm{median}\left({R}_{t,k}\right) $$

$$ \mathrm{If}\ {P}_{\left(t,k\right)}>16,\mathrm{then}\ {P}_{\left(t,k\right)}=16 $$

Thereafter, the profiles are partitioned into segments of similar read depth ratios, and the copy number status of a segment are determined by the average read depth of probes within the segment. After that, adjacent segments are merged hierarchically to form a larger CNV candidate if they have the same copy number status.

However, it is difficult to detect small size changes using the above CBS. To address this issue, we added duplication or deletion regions covered by two or more consecutive strong probe-level CNV signals to increase the sensitivity of our method. For each CNV candidate generated from the above, its copy number and CNV length are calculated. We estimated the copy number by the average of the copy numbers of probes inferred from their read-depth ratio. Because the exact breakpoints of CNV candidates cannot be determined with DeviCNV, the start/end genomic position or length of the CNV candidates are annotated based on the probe information provided by the user. Additionally, DeviCNV annotates the CNV type, sample name, and median of reads depth of each probe/primer pool, the genomic position of the CNV candidate, and confidence information for the predicted reads depth ratios supporting the candidate.

### Scoring CNVs

To detect CNVs with high specificity, DeviCNV evaluates all CNV candidates using the following five scoring criteria (Table 3 and Additional file 1: Note S12) to determine confidence levels. To define the thresholds or condition for each criterion, we used the IMD_HYB dataset and the IMD_PCR dataset from eight cell lines with known CNVs. The five scoring criteria are as followed: 1) *ProbeCntInRegion:* the number of probes within the CNV candidate, 2) *AverageOfReadDepthRatios:* the average of reads depth ratios of probes within the CNV candidate, 3) S*TDOfReadDepthRatios:* the standard deviation of the read depth ratio of the probes within the CNV candidate, 4) *AverageOfCIs*: The average length of 95% confidence interval of read-depth ratios of the probes within the CNV candidate, and 5) *AverageOfR2vals:* the average of average R-squared values of the linear regression models for probes within the CNV candidate. If a CNV candidate passes each criterion, one point is assigned; then, the CNV candidates that scored 5 points are designated as final CNV candidates. More detailed descriptions of the threshold for each criterion are provided in Additional file 1: Note S12.

### Visualization

**Fig. 3:**
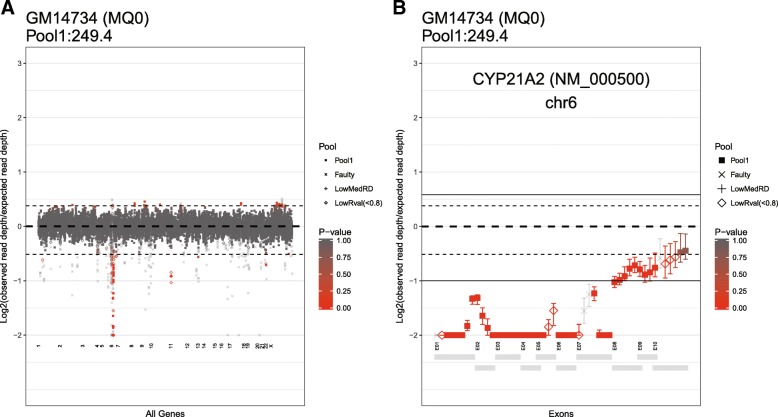
Example of DeviCNV plots. Predicted read-depth ratios (observed read depth/predicted read depth) of probes on a panel plotted on a log_2_ scale for each sample: **a** the whole-genome view plot depicts all probes on a panel, and **b** the gene-centric view plot depicts the probes within a gene. Each point represents the read-depth ratio for each probe, and its shape indicates the pool or an assessment of faulty or low-quality types that are classified when building the linear regression models. The color of each point shows the p-value for duplications and deletions (the thresholds are set at 1.3 and 0.7, thin black dotted lines). The whiskers represent the 95% confidence interval for the read-depth ratio. This is an example of a multi-exon deletion within *CYP21A2* found in a cell line using the inherited metabolic disorder panel and the hybridization capture approach

DeviCNV allows visualization of CNV results as graphical plots with predicted read-depth ratios. There are two types of plots: a whole-genome view plot for the whole gene showing the overall result for one sample across whole genes (Fig. 3a), and the gene-centric view plots containing detailed information (Fig. 3b). In the plot, grey dotted lines indicate duplication/deletion thresholds. The shape of points in the plot indicates different primer/probe pool and if the probes are faulty or low-quality. The red-white gradient indicates the *p*-value which is defined by 1 − *p*. *dup*_(*t*, *k*)_ or 1 − *p*. *del*_(*t*, *k*)_ for a given target probe t in the k sample. A 95% confidence interval for the predicted read-depth ratio is also displayed that indicates the reliability of each result. By displaying various parameters on this graph, users can check the results directly and easily.

### Generation of targeted NGS datasets

We evaluated DeviCNV using four targeted NGS datasets sequenced for use in clinical research (Table 1). First, we used our IMD (inherited metabolism disorders) gene panels that were developed using two different capture approaches: hybridization-based capture (IMD_HYB) and PCR-based capture (IMD_PCR) (Additional file 1: Note S13). The IMD_HYB panel consisted of 19,210 probes. The IMD_PCR panel consisted of 9072 amplicons separated into three pools to prevent reactions between primers. We sequenced targeted NGS data derived from both IMD_HYB and IMD_PCR capture assays, followed by sequencing using HiSeq (Illumina, San Diego, CA, USA) and Ion S5 (Thermo Fisher Scientific, Waltham, MA, USA) platforms. We sequenced a total of 96 targeted NGS datasets from 72 unique samples (27 cell lines and 45 clinical samples). Secondly, we used our previous version of the IMD panel, IMD_V1, developed using only for the PCR-based capture method. This panel consists of 2054 amplicons in two pools, and a total of 178 clinical datasets were sequenced using the Ion Torrent Personal Genome Machine (PGM) system (Thermo Fisher Scientific, Waltham, MA, USA).

For each sample data sequenced using the hybridization-based method, the targeted NGS data were aligned to the human reference genome (hs37d5) using BWA 0.7.12 [33], Picard 1.139 tools (http://broadinstitute.github.io/picard/) were applied to sort and mark duplicated reads, and the Genome Analysis Toolkit (GATK) 3.4.46 [34] was applied for recalibration and indel realignment, according to the GATK Best Practices guidelines [35]. The data sequenced using the PCR-based approach were processed with standard Ion Torrent Suite™ Software, and the Torrent Server was used for alignment (Additional file 1: Note S14).

### Running parameters of other tools for the performance comparison

For VisCap, we set *iqr_multiplier* at 1.1 and *threshold.cnv_log2_cutoffs* at (log_2_ [0.7], log_2_ [1.3]) to maximize sensitivity because our DeviCNV parameters were set for maximum sensitivity detection, whereas, for other parameters, the default settings were used. In addition, we ran VisCap with default parameters. We used ‘run_1’ results, which were analyzed without sample QC filtering of VisCap because sample failure rates of ‘run_2’ were too large to analyze (Additional file 1: Note S15).

For the XHMM QC and filtering step, we set the parameters so that XHMM performed best for our data. To remove the gender-specific effect of the X chromosome, we used the normalized depth of coverage data by dividing the number of X chromosomes in samples from females in half. During the *Filters samples and targets and then mean-centers the targets* step, we set the *maxSdSampleRD* to 400, the *minMeanTargetRD* to 50, and the *minMeanSampleRD* to 50*.* For the *Filters and z-score centers (by sample) the PCA-normalized data* step, *maxSdTargetRD* was set to 400 instead of 30. Then, in the *Discovers CNVs in normalized data* step, we set *mean number of targets in CNV* to 2 and used default settings for other parameters.

For CODEX, we ran targeted sequencing with default parameter settings for the QC and CNV calling steps.

## Additional file

Additional file 1: **Note S1.** Performance comparison based on the mean target depth for a sample. **Note S2.** Performance evaluation of DeviCNV by qPCR. **Note S3.** Performance comparison to VisCap, XHMM, and CODEX. **Note S4.** Sample collection description of for the inherited metabolic disorder panel. **Note S5.** Visual inspection process to find pathogenic CNVs in patients. **Note S6.** Performance comparison based on the number of input samples. **Note S7.** Performance comparison based on the configuration of the sample set used as an input. **Note S8.** Differences in the number of data points for each exon based on input intervals. **Note S9.** Performance comparison based on MQV thresholds. **Note S10.** Low-quality sample filter by using sample-to-sample correlation. **Note S11.** Performance comparison based on duplication and deletion thresholds for read depth ratios. **Note S12.** Unique scoring system for selecting high-confidence CNV candidates. **Note S13.** Inherited metabolic disorder (IMD) panel description. **Note S14.** Generating targeted NGS data. Note S15. Failure rate of DeviCNV, VisCap, XHMM, and CODEX. **Note S16.** List of abbreviations. (PDF 908 kb)

## Abbreviations

BAM: Binary alignment/map
Bp: Base pairs
CBS: Circular binary segmentation
CI: Confidence interval
CN: Copy number
CNV: Copy number variant
DEL: Deletion
DUP: Duplication
GATK: Genome Analysis Toolkit
HC: Hereditary cancer
IMD: Inherited metabolism disorders
INDEL: Short insertion and deletion
MQV: Mapping quality value
NEU: Neutral
NGS: Next generation sequencing
PCA: Principal component analysis
PCR: polymerase chain reaction
PGM: The Ion Torrent Personal Genome Machine
QC: Quality control
qPCR: Quantitative polymerase chain reaction
SNV: Single-nucleotide variant
