# Supplementary material for: DeviCNV: detection and visualization of exon-level copy number variants in targeted next-generation sequencing data
Source: BMC Bioinformatics. 2018 Oct 16;19:381. doi: 10.1186/s12859-018-2409-6 (PMC6192323; doi:10.1186/s12859-018-2409-6)
Supplement: Supplementary file 1 — Note S1. Performance comparison based on the mean target depth for a sample. Note S2. Performance evaluation of DeviCNV by qPCR. Note S3. Performance comparison to VisCap, XHMM, and CODEX. Note S4. Sample collection description of for the inherited metabolic disorder panel. Note S5. Visual inspection process to find pathogenic CNVs in patients. Note S6. Performance comparison based on the number of input samples. Note S7. Performance comparison based on the configuration of the sample set used as an input. Note S8. Differences in the number of data points for each exon based on input intervals. Note S9. Performance comparison based on MQV thresholds. Note S10. Low-quality sample filter by using sample-to-sample correlation. Note S11. Performance comparison based on duplication and deletion thresholds for read depth ratios. Note S12. Unique scoring system for selecting high-confidence CNV candidates. Note S13. Inherited metabolic disorder (IMD) panel description. Note S14. Generating targeted NGS data. Note S15. Failure rate of DeviCNV, VisCap, XHMM, and CODEX. Note S16. List of abbreviations. (PDF 908 kb) [file 12859_2018_2409_MOESM1_ESM.pdf]

Supplementary information for the article

DeviCNV: Detection and Visualization of Exon-Level Copy Number Variants in Targeted Next-Generation Sequencing Data

Yeeok Kang[1,2], Seong-Hyeuk Nam[1], Kyung Sun Park[1], Yoonjung Kim[3], Jong-Won Kim[4], Eunjung Lee[5], Jung Min Ko[6], Kyung-A Lee[3,*], Inho Park[1,*]

[1]SD Genomics Co., Ltd., 11F, Seoul Gangnam Post Office, 619 Gaepo-ro, Gangnam-gu, Seoul 06336, Republic of Korea; [2]Department of Bio and Brain Engineering, KAIST, 291 Daehak-ro, Yuseong-gu, Daejeon, Republic of Korea; [3]Department of Laboratory Medicine, Yonsei University College of Medicine, 211 Eonjuro, Gangnam-gu, Seoul 06273, Republic of Korea; [4]Department of Laboratory Medicine and Genetics, Samsung Medical Center, Sungkyunkwan University School of Medicine, Seoul, Republic of Korea; [5]Division of Genetics and Genomics, Boston Children's Hospital and Harvard Medical School, Boston, USA; [6]Department of Pediatrics, Seoul National University Children's Hospital, Seoul National University College of Medicine, Seoul, Republic of Korea

*corresponding author

Note S1. Performance comparison based on the mean target depth for a sample

To identify the minimum mean target depth in samples for detecting real CNVs, we ran DeviCNV with down-sampled samples. We selected one sample in a batch and made 10 input sets by down-sampling it by uisng samtools [1]. Total 40 input sets were created by repeating this process for four samples. We ran DeviCNV using these 40 sets as inputs. We examined whether the known CNVs were found in these down-sampled samples for each input set, and the number of detected candidates was measured.

In NA06804, known CNVs were not found when the mean target depth dropped to below 100X. Similarly, mean target depth dropped to below 60X for NA22208 sample, so no known CNV was detected. In the case of NA01741, the known CNV was well-detected regardless of how low the mean target depth was for the sample. These samples assumed that they have good uniformity and strong CNV signal. Likewise, the lower the mean target depth, the more the number of CNV candidates that were selected. Lastly, in GM14603, which had low uniformity, the number of CNV candidates did not change according to its mean target depth. As a result, this sample showed too many CNV candidates. In the case of IMD_PCR GM14603, similarly to IMD_HYB samples, the read depth of each pool dropped below 100, so no known CNV was detected.

Panel& Platform	Sample	Gene	NM	CNV	CNV size (kb)	Down sampling ratio	mean target depth	Median read depth	Find?[a]	#CNV[b]
IMD_HYB& DeviCNV_HYB	NA06804	*HPRT1*	NM_000194	EX2-3 DUP	2.01	1	242X	262	O	39
						0.9	218X	235.7	O	27
						0.8	194X	209.9	O	19
						0.7	169X	183.4	O	20
						0.6	145X	157.1	O	23
						0.5	121X	130.9	O	47
						0.4	97X	104.7	O	156
						0.3	73X	78.3	X	146
						0.2	48X	52.1	X	12
						0.1	24X	25.8	X	23
	NA22208	*PCCA*	NM_000282	EX13-20 DEL	146.38	1	184X	199.6	O	3
						0.9	166X	179.5	O	3
						0.8	147X	159.4	O	2
						0.7	129X	139.4	O	4
						0.6	110X	119.4	O	20
						0.5	92X	99.2	A	41
						0.4	74X	79.4	A	73
						0.3	55X	59.4	X	28
						0.2	37X	39.4	X	1
						0.1	18X	19.5	X	44
IMD_PCR& DeviCNV_PCR	NA01741	*GALT*	NM_000155	Entire gene DEL	4.01	1	524X	Pool 1: 408.0, Pool 2: 556.0, Pool 3: 271.0	O	12
						0.9	472X	Pool 1: 365.0, Pool 2: 501.0, Pool 3: 243.0	O	12
						0.8	419X	Pool 1: 324.0, Pool 2: 444.0, Pool 3: 216.0	O	14
						0.7	367X	Pool 1: 285.0, Pool 2: 389.0, Pool 3: 189.0	O	22
						0.6	314X	Pool 1: 244.0, Pool 2: 333.0, Pool 3: 162.0	O	30
						0.5	262X	Pool 1: 203.0, Pool 2: 278.0, Pool 3: 135.0	O	34
						0.4	210X	Pool 1: 162.5, Pool 2: 220.0, Pool 3: 108.0	O	49
						0.3	157X	Pool 1: 122.0, Pool 2: 166.0, Pool 3: 80.0	O	23
						0.2	105X	Pool 1: 81.5, Pool 2: 110.0, Pool 3: 53.0	O	3
						0.1	52X	Pool 1: 41.0, Pool 2: 55.0, Pool 3: 27.0	O	1
	GM14603	*GAA*	NM_000152	EX18 DEL	0.16	1	197X	Pool 1: 215.0, Pool 2: 141.0, Pool 3: 90.0	O	24
						0.9	177X	Pool 1: 193.0, Pool 2: 126.0, Pool 3: 81.0	O	17

						0.8	157X	Pool 1: 172.0, Pool 2: 112.0, Pool 3: 72.0	O	10
						0.7	138X	Pool 1: 150.0, Pool 2: 98.0, Pool 3: 63.0	X	4
						0.6	118X	Pool 1: 129.0, Pool 2: 84.0, Pool 3: 54.0	X	1
						0.5	98X	Pool 1: 108.0, Pool 2: 70.0, Pool 3: 45.0	X	0
						0.4	79X	Pool 1: 85.0, Pool 2: 56.0, Pool 3: 36.0	X	0
						0.3	59X	Pool 1: 64.0, Pool 2: 42.0, Pool 3: 28.0	X	0
						0.2	39X	Pool 1: 42.5, Pool 2: 28.0, Pool 3: 18.0	X	0
						0.1	20X	Pool 1: 21.0, Pool 2: 14.0, Pool 3: 9.0	X	3

CNV, copy number variation; IMD, inherited metabolism disorder; HYB, hybridization-based capture approach; PCR, polymerase chain reaction based capture approach; EX, exon; DEL, deletion; DUP, duplication.

[a]Indicates whether each method found a known CNV. "O" means all CNVs were found, "X" means they were not found at all, and "A" means they were found only in some exons; [b]indicates the number of CNV candidates that received the highest score of 5 found in the corresponding sample.

Note S2. Performance evaluation of DeviCNV by qPCR

DeviCNV detects raw CNV candidates, and then assigns scores to CNV candidates via the scoring system: 5-score CNV candidates, 4-score CNV candidates, 3-score CNV candidates, 2-score CNV candidates, 1-score CNV candidates and 0-score CNV candidates.

To evaluate the performance of DeviCNV, we performed qPCR on the subset of CNV candidates with confidence score of 5 from the IMD_HYB dataset. The subset was selected from 11 cell lines with the number of CNV candidates of score 5 less than 10, which resulted in a total of 40 CNV candidates (27 duplications and 13 deletions). We randomly selected 25 of the 497 CNV candidates with confidence score of 4 from the above 11 cell lines. We selected 25 CNV candidates in 10 samples.

The table below shows the information and confirmation results of selected CNV candidates.

Score	Type	CN	Sample	Chr	Start	End	CNV size (kb)	Gene	NM	Exons	Confirmed by qPCR
5	del	-2	GM14734	6	32006027	32008071	2.05	*CYP21A2*	NM_000500	Exon1-7	Known CNV
5	del	-1	GM14734	6	32008112	32008981	0.87	*CYP21A2*	NM_000500	Exon8-10	Known CNV
5	del	-1	GM17433	9	6595003	6595183	0.18	*GLDC*	NM_000170	Exon9	Failed
5	dup	1	GM17433	11	68552392	68552572	0.18	*CPT1A*	NM_001876	Exon10	Confirmed
5	del	-1	GM17433	11	118179071	118179251	0.18	*CD3E*	NM_000733	Exon4	Confirmed
5	dup	1	GM17433	14	64879149	64879299	0.15	*MTHFD1*	NM_005956	Exon4	Failed
5	del	-1	GM17433	14	94849098	94849278	0.18	*SERPINA1*	NM_000295	Exon2	Failed
5	dup	1	GM17433	15	45654339	45654476	0.14	*GATM*	NM_001482	Exon9	Confirmed
5	dup	1	GM17433	19	17948790	17948912	0.12	*JAK3*	NM_000215	Exon12	Failed
5	dup	1	GM24007	1	198697518	198698375	0.86	*PTPRC*	NM_002838	Exon16-17	Confirmed
5	dup	1	GM24007	3	81548310	81548446	0.14	*GBE1*	NM_000158	Exon15	Failed
5	dup	1	GM24007	6	70410646	70410826	0.18	*LMBRD1*	NM_018368	Exon12	Confirmed
5	dup	1	GM24007	12	21007891	21008131	0.24	*SLCO1B3*	NM_019844	Exon4	Confirmed
5	del	-1	GM24007	12	103260303	103260511	0.21	*PAH*	NM_000277	Exon5	Confirmed

5	del	-1	GM24007	X	30322625	30327562	4.94	*NR0B1*	NM_000475	Exon1-2	Partially confirmed (EX1)
5	del	-1	GM24007	X	38211879	38280405	68.53	*OTC*	NM_000531	Exon1-10	Known CNV
5	del	-1	NA00006	11	108002563	108002743	0.18	*ACAT1*	NM_000019	Exon2	Failed
5	dup	1	NA00852	2	211504649	211504853	0.21	*CPS1*	NM_001875	Exon24	Failed
5	dup	1	NA00852	6	32006027	32006628	0.60	*CYP21A2*	NM_000500	Exon1-2	Failed
5	dup	1	NA00852	6	32008232	32008832	0.60	*CYP21A2*	NM_000500	Exon8-10	Failed
5	del	-1	NA00852	16	223233	223771	0.54	*HBA2*	NM_000517	Exon2-3	Confirmed
5	dup	1	NA01741	1	120295929	120296072	0.14	*HMGCS2*	NM_005518	Exon7	Failed
5	dup	1	NA01741	8	133879175	133880409	1.24	*TG*	NM_003235	Exon1-2	Failed
5	dup	1	NA01741	8	133925221	133925401	0.18	*TG*	NM_003235	Exon20	Confirmed
5	dup	1	NA01741	8	133931550	133931730	0.18	*TG*	NM_003235	Exon21	Confirmed
5	del	-2	NA01741	9	34646504	34650535	4.03	*GALT*	NM_000155	Exon1-11	Known CNV
5	dup	1	NA01741	15	45389861	45390009	0.15	*DUOX2*	NM_014080	Exon28	Failed
5	dup	1	NA02227	2	211456544	211456784	0.24	*CPS1*	NM_001875	Exon10	Failed
5	dup	1	NA02227	6	32008772	32008981	0.21	*CYP21A2*	NM_000500	Exon10	Confirmed
5	dup	1	NA02227	10	56076960	56077200	0.24	*PCDH15*	NM_033056	Exon8	Failed
5	del	-1	NA02659	2	211521178	211521358	0.18	*CPS1*	NM_001875	Exon30	Failed
5	del	-1	NA02659	16	223400	223771	0.37	*HBA2*	NM_000517	Exon3	Confirmed
5	dup	1	NA11781	1	155207974	155208154	0.18	*GBA*	NM_001005741	Exon7	Failed
5	dup	1	NA12217	1	155204715	155205521	0.81	*GBA*	NM_001005741	Exon10-12	Confirmed
5	dup	1	NA22496	7	65429359	65429539	0.18	*GUSB*	NM_000181	Exon11	Confirmed
5	dup	1	NA22496	8	134128842	134129022	0.18	*TG*	NM_003235	Exon45	Failed
5	dup	1	NA22496	14	94847137	94847317	0.18	*SERPINA1*	NM_000295	Exon3	Failed
5	dup	1	NA22496	17	41055977	41056117	0.14	*G6PC*	NM_000151	Exon2	Confirmed
5	dup	1	NA22496	X	18947275	18947515	0.24	*PHKA2*	NM_000292	Exon13	Failed
5	dup	1	NA22496	X	48652429	48652669	0.24	*GATA1*	NM_002049	Exon6	Failed
4	del	-1	GM17433	5	78076405	78076569	0.17	*ARSB*	NM_000046	Exon8	Failed
4	dup	1	GM17433	22	31019004	31019140	0.14	*TCN2*	NM_000355	Exon8	Confirmed
4	dup	1	GM23221	8	133947930	133948170	0.24	*TG*	NM_003235	Exon25	Failed
4	del	-1	GM23221	X	71822990	71823138	0.15	*PHKA1*	NM_002637	Exon26	Confirmed
4	dup	1	GM24007	6	70411710	70411915	0.21	*LMBRD1*	NM_018368	Exon10	Failed
4	dup	1	GM24007	7	117251782	117251924	0.14	*CFTR*	NM_000492	Exon20	Failed
4	del	-1	NA00006	12	110011081	110011381	0.30	*MMAB*	NM_052845	Exon1	Failed
4	del	-1	NA00852	16	222841	223771	0.93	HBA2	NM_000517	Exon1-3	Confirmed
4	dup	1	NA00852	X	71904277	71904517	0.24	*PHKA1*	NM_002637	Exon5	Failed
4	del	-1	NA01741	1	198608328	198608568	0.24	*PTPRC*	NM_002838	Exon2	Failed
4	dup	1	NA01741	8	134145763	134146003	0.24	*TG*	NM_003235	Exon47	Failed
4	dup	1	NA01741	11	76919768	76922452	2.69	*MYO7A*	NM_000260	Exon44-45	Partially confirmed (EX44)
4	dup	1	NA01741	X	48652429	48652669	0.24	*GATA1*	NM_002049	Exon6	Failed
4	dup	1	NA02227	2	211440999	211441299	0.30	*CPS1*	NM_001875	Exon3	Confirmed
4	dup	1	NA02227	3	182788716	182789215	0.50	*MCCC1*	NM_020166	Exon6-7	Failed
4	dup	1	NA02227	5	41739421	41739661	0.24	*OXCT1*	NM_000436	Exon16	Failed
4	del	-1	NA02227	X	18969151	18969451	0.30	*PHKA2*	NM_000292	Exon4	Failed
4	dup	1	NA02227	X	77258559	77258799	0.24	*ATP7A*	NM_000052	Exon6	Confirmed
4	del	-1	NA02659	14	94847197	94847518	0.32	*SERPINA1*	NM_000295	Exon3	Failed

4	del	-1	NA11781	1	119957972	119962270	4.30	*HSD3B2*	NM_000198	Exon2-3	Failed
4	del	-1	NA11781	1	119964541	119965321	0.78	*HSD3B2*	NM_000198	Exon4	Confirmed
4	dup	1	NA11781	6	32008292	32008532	0.24	*CYP21A2*	NM_000500	Exon8-9	Confirmed
4	dup	1	NA11781	11	76858893	76859073	0.18	*MYO7A*	NM_000260	Exon4	Failed
4	dup	1	NA11781	19	41919883	41920123	0.24	*BCKDHA*	NM_000709	Exon4	Confirmed
4	dup	1	NA22496	X	18944680	18947515	2.84	*PHKA2*	NM_000292	Exon13-14	Failed

CN, copy number; Chr, chromosome; CNV, copy number variation; DEL, deletion; DUP, duplication.

Note S3. Performance comparison to VisCap, XHMM, and CODEX

According to qPCR confirmation, total 16 CNVs with confidence score of 5 detected from DeviCNV were confirmed (See Note S2). We evaluated how many of these CNVs could be detected with other tools: VisCap, XHMM, and CODEX.

The table below shows the information and detection results of the qPCR CNVs.

Type	Sample	Chr	Start	End	CNV size (kb)	Gene	NM	Exon	DeviCNV	VisCap	XHMM	CODEX
DUP	GM17433	11	68552283	68552478	0.20	*CPT1A*	NM_001876	Exon10	O	X	X	X
DEL	GM17433	11	118179142	118179156	0.01	*CD3E*	NM_000733	Exon04	O	X	X	O
DUP	GM17433	15	45653322	45654419	1.10	*GATM*	NM_001482	Exon09	O	X	X	X
DUP	GM24007	1	198697469	198698300	0.83	*PTPRC*	NM_002838	Exon16-17	O	X	X	O
DUP	GM24007	6	70410657	70410761	0.10	*LMBRD1*	NM_018368	Exon12	O	X	X	X
DUP	GM24007	12	21007962	21008103	0.14	*SLCO1B3*	NM_019844	Exon04	O	X	X	O
DEL	GM24007	12	103260374	103260441	0.07	*PAH*	NM_000277	Exon05	O	O	X	X
DEL	GM24007	X	30326313	30327495	1.18	*NR0B1*	NM_000475	Exon01	O	O	X	O
DEL	NA00852	16	223124	223709	0.59	*HBA2*	NM_000517	Exon02-03	O	X	O	X
DUP	NA01741	8	133925292	133925510	0.22	*TG*	NM_003235	Exon20	O	X	X	X
DUP	NA01741	8	133931621	133931770	0.15	*TG*	NM_003235	Exon21	O	X	X	X
DUP	NA02227	6	32008646	32009447	0.80	*CYP21A2*	NM_000500	Exon10	O	X	X	X
DEL	NA02659	16	223471	223709	0.24	*HBA2*	NM_000517	Exon03	O	X	O	X
DUP	NA12217	1	155204239	155205102	0.86	*GBA*	NM_001005741	Exon12-11	O	X	X	X
DUP	NA22496	7	65429310	65429445	0.14	*GUSB*	NM_000181	Exon11	O	X	X	X
DUP	NA22496	17	41055948	41056057	0.11	*G6PC*	NM_000151	Exon02	O	X	X	O

Chr, chromosome; CNV, copy number variation; DEL, deletion; DUP, duplication.

[a]Indicates whether each method found a known CNV. "O" means CNVs were found, and "X" means they were not found at all.

Note S4. Sample collection description of for the inherited metabolic disorder panel

Inclusion criteria

(1) Patients who have been commissioned by primary/secondary hospitals or Seoul National University Children's Hospital newborn room to department of Pediatrics in SNU Children's Hospital for additional confirmation after abnormal findings in Inherited metabolic screening, which is performed on all newborns in Korea.

(2) Patients currently in treatment, diagnosed from secondary blood/urine metabolism testing without identified genetic cause, after abnormal findings in initial IMD screening in Korea.

Exclusion criteria

(1) Low birth weight less than 2.0 kg

(2) Premature infants less than 35 weeks

Note S5. Visual inspection process to find pathogenic CNVs in patients

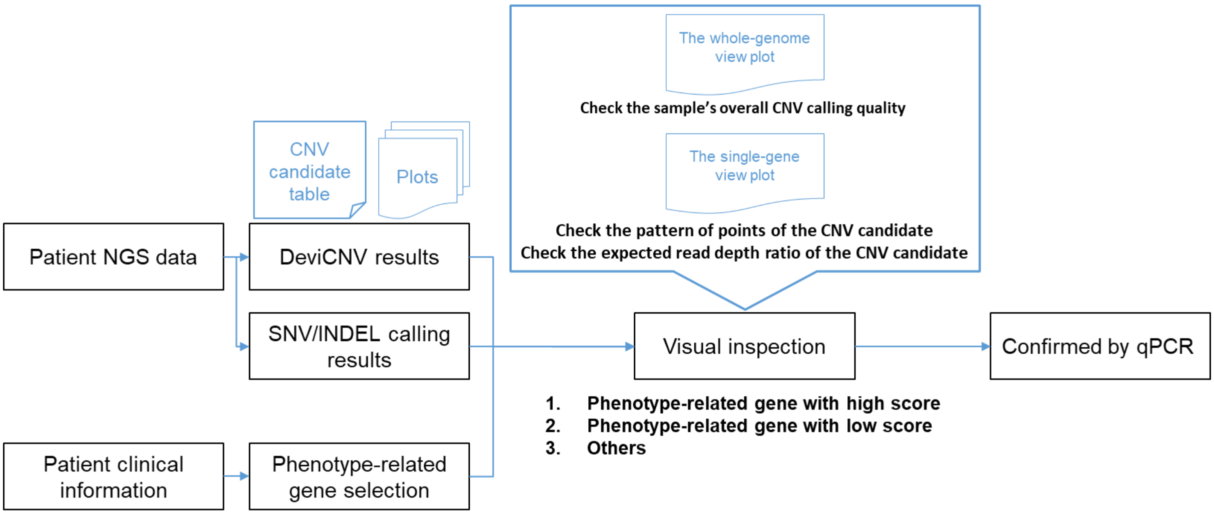

DeviCNV was used to find pathogenic CNVs in patients with inherited metabolic disorders through the IMD panel.

First, a CNV candidate list table and plots (one whole-genome view plot and single-gene view plots) were generated by DeviCNV, and SNV/INDEL analysis results were also obtained using SNV/INDEL caller with patient NGS data. We also selected genes related to patient phenotype by analyzing clinical information of patients. By combining these three sets of information, our clinician selected pathogenic CNV through visual inspection. The order of inspecting CNV candidates was as follows: (1) within phenotype-related gene and with high score, (2) within phenotype-related gene and with low score, and (3) others. To perform the visual inspection, the clinician first looked at the overall CNV calling quality through the whole-genome view plot of the sample. The clinician checked that the mean target depth of the sample was not too low, how many data points were classified as low-quality type, and that there were not too many duplications or deletions. Next, the clinician

looked at the gene-centric view plot with the CNV candidate. The clinician identified patterns of data points belonging to the CNV candidate, and confirmed that the expected read depth ratio was sufficiently low or increased.

Through this process, the clinician selected pathogenic CNV candidates and confirmed them by qPCR.

Note S6. Performance comparison based on the number of input samples

To find the minimum number of input samples to run DeviCNV, nine experiments were performed with input sample sets that consist of five to 13 samples. For all experiments, all parameters were default setting. Failure rates of all experiments were 0%.

When five input samples were input to DeviCNV, no linear regression model was created for all probes, and no results were generated. Therefore, at least six samples are required. In addition, we checked whether or not each experimental setup detected eight known cell-line CNVs, and how many CNV candidates with confidence score of 5 were detected for each sample.

Sample			CNV				The number of input samples								
Panel	Cell line	Median read depth	Gene	NM	CNV	CNV size (kb)	5	6	7	8	9	10	11	12	13
IMD_HYB	GM14603	81.99	*GAA*	NM_000152	EX18 DEL	0.16	NA	X[a] (0)[b]	X (0)	X (0)	X (5)	X (2)	X (1)	X (1)	X (3)
	GM14734	249.4	*CYP21A2*	NM_000500	30 KB DEL, Entire gene DEL	3.35	NA	O (1)	X (0)	O (3)	O (4)	O (3)	O (2)	O (3)	O (4)
	GM24007	142.84	*OTC*	NM_000531	Entire gene DEL	68.97	NA	X (0)	O (1)	O (3)	O (4)	O (6)	O (9)	O (10)	O (9)
	NA01741	164.4	*GALT*	NM_000155	Entire gene DEL	4.01	NA	X (3)	O (1)	O (6)	O (9)	O (7)	O (11)	O (14)	O (14)
	NA06804	261.98	*HPRT1*	NM_000194	EX2-3 DUP	2.01	NA	X (7)	O (24)	O (21)	O (23)	O (33)	O (34)	O (46)	O (40)
	NA06805	80.13	*GALC*	NM_000153	EX11-17 DEL	17.73	-	X (1)	X (1)	X (1)	O (16)	O (11)	O (7)	O (5)	O (5)
	NA12217	269.08	*CYP21A2*	NM_000500	30 KB DEL	1.14	-	-	X (0)	X (1)	X (1)	X (2)	X (4)	X (3)	O (7)
	NA22208	199.64	*PCCA*	NM_000282	EX13-20 DEL	146.38	-	-	-	O (3)	O (6)	O (9)	O (7)	O (9)	O (14)

CNV, copy number variation; IMD, inherited metabolism disorder; HYB, hybridization based capture approach; EX, exon; DEL, deletion; DUP, duplication; NA, not available.

[a]Indicates whether each method found a known CNV. "O" means all CNVs were found, and "X" means they were not found at all; [b]indicates the number of CNV candidates that received the highest score of 5 found in the corresponding sample.

Note S7. Performance comparison based on the configuration of the sample set used as an input

We recommend that the input should consist of a sample set from the same batch. We compared CNV detection performance when using an input set obtained by mixing samples from different batches with when using an input set with samples from the same batch.

We used three batch sets of data from the IMD_HYB: original_batch1, original_batch2 and original_batch3. We randomly mixed 66 samples to construct three random batch sets: random_batch1, random_batch2 and random_batch3. We also combined all the samples into a single set without regard to their batch: “merged all” batch.

Each set was analyzed using DeviCNV_HYB. Of 66 samples, seven samples in the original batch, 10 samples in the random batch and 10 samples in the combined batch were filtered out, due to a low correlation coefficient. For comparisons under the same conditions, the mean and median of the raw and highest confidence CNV candidates were measured, except for the samples that were filtered out in any of the three experiments.

The following table shows statistical values for raw CNV candidates before scoring.

#raw CNV	Original batch			Random batch			“Merged all” batch		
	DUP	DEL	TOTAL	DUP	DEL	TOTAL	DUP	DEL	TOTAL
Average	48.7	23.1	71.8	103.5	50.3	153.8	116.9	53.6	170.5
Median	13	6	22	48	31	88	48.5	42.5	98.5

Average, an average of raw CNV candidates of 56 samples; Median, a median of raw CNV candidates of 56 samples; DEL, deletion; DUP, duplication; TOTAL, the total number of deletions and duplications.

The following table shows statistical values for CNV candidates with confidence score of 5, which is the highest score from the scoring system of DeviCNV.

#CNV	Original batch			Random batch			“Merged all” batch		
	DUP	DEL	TOTAL	DUP	DEL	TOTAL	DUP	DEL	TOTAL
Average	5.8	2.3	8.1	16.9	5.8	22.7	21.9	8.3	30.2
Median	1	0	2	5	2	9.5	8.5	2	13.5

Average, an average of 5-score CNV candidates of 56 samples; Median, a median of 5-score CNV candidates of 56 samples; DEL, deletion; DUP, duplication; TOTAL, the total number of deletions and duplications.

In experiments using the original batch, a small number of CNVs was detected. In contrast, the largest number of CNVs was detected from experiments using the random batch and “merged all” batch.

For the "merged all" batch experiment, the number of input samples was three times that of the random batch experiment. Since there were more data to use for modeling, we expected that performance should improve and detect fewer CNV candidates. Alternatively, in both experiments, we expected that CNV candidates should be found in similar numbers since the proportion of samples from the same batch was the same in the input sample set configuration. However, we observed less CNV candidates in the random batch experiment than in the "merged all" batch experiment.

To analyze this, we plotted the median read depth of the sample as X value, and the number of raw CNV candidates found in the "merged all" batch minus the number of raw CNV candidates found in the random batch as the Y value. As a result, we found that the samples with low median read depth (<200X) weirdly got more CNV candidates in the "merged all" batch. Therefore, users need to look carefully at the results when calling the CNV of a sample with depth less than 200.

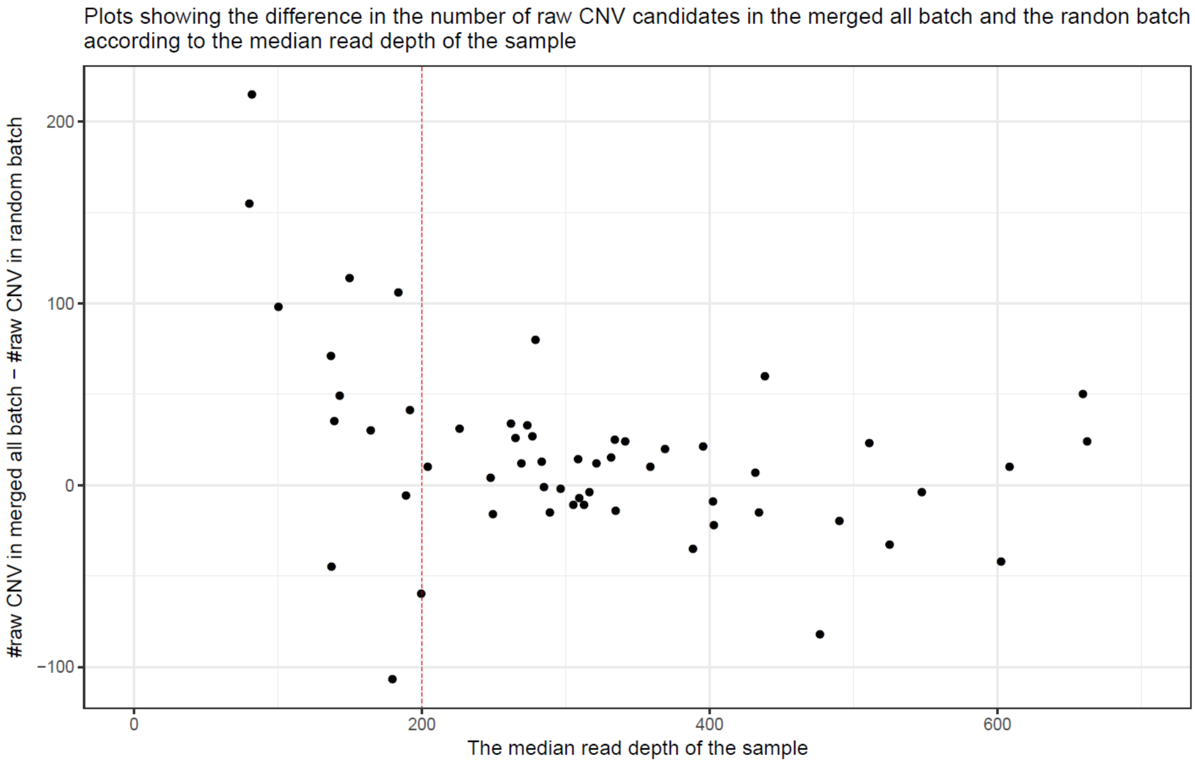

In DeviCNV_HYB samples, eight cell-line samples had known CNVs. We checked whether each experiment detected the eight known CNVs or not, and how many CNV candidates were detected for each sample. As a result, seven known CNVs were detected in three experiments. The numbers of CNV candidates with confidence score of 5 found in each

cell-line sample are shown in the following table. Usually the larger the median read depth of the sample is, the fewer candidates are detected.

Sample			Known CNV				Original Batch		Random Batch		"Merged all" Batch	
Panel	Cell line	Median read depth	Gene	NM	CNV	CNV size (kb)	Find?[a]	#CNV[b]	Find?	#CNV	Find?	#CNV
IMD_HYB	GM14603	81.99	*GAA*	NM_000152	EX18 DEL	0.16	O	24	X	4	X	8
	GM14734	249.4	*CYP21A2*	NM_000500	30 KB DEL, Entire gene DEL	3.35	O	2	O	29	O	37
	GM24007	142.84	*OTC*	NM_000531	Entire gene DEL	68.97	O	7	O	10	O	12
	NA01741	164.4	*GALT*	NM_000155	Entire gene DEL	4.01	O	6	O	20	O	28
	NA06804	261.98	*HPRT1*	NM_000194	EX2-3 DUP	2.01	O	34	O	56	O	63
	NA06805	80.13	*GALC*	NM_000153	EX11-17 DEL	17.73	O	44	O	11	O	16
	NA12217	269.08	*CYP21A2*	NM_000500	30 KB DEL	1.14	X	1	O	9	O	15
	NA22208	199.64	*PCCA*	NM_000282	EX13-20 DEL	146.38	O	3	O	5	O	12

CNV, copy number variant; EX, exon; DEL, deletion; DUP, duplication.

[a]Indicates whether each method found a known CNV. "O" means all CNVs were found, and "X" means they were not found at all; [b]indicates the number of CNV candidates that received the highest score of 5 found in the corresponding sample.

Note S8. Differences in the number of data points for each exon based on input intervals

Usually, one more probes tiled in one exon for targeted NGS panel design. When we analyze NGS data, we considered which unit is better to detect CNV signal: individual probes or individual exons (or merged probes).

DeviCNV does not merge overlapping probe (or amplicon) intervals, which would cause reduction in probe-specific information; instead, it analyzes probes separately. Therefore, a probe-level read depth is estimated and a probe-level read depth ratio is predicted, and DeviCNV can obtain one more signals for one exon.

In contrast, if a merged probe interval is used for a single exon or a target interval (usually an exon interval) for one exon, usually only one signal can be collected for each exon. In the case of fewer data points per exon, it is not likely to detect single exon CNVs; and even if some CNVs are detected, they would be less reliable.

To confirm differences of detecting performances between using individual probes and merged probes (=target intervals), we ran DeviCNV to analyze IMD_HYB and IMD_PCR samples with probes and targets(exon) as interval inputs.

In the experiment with probes as the interval input, we used default parameter setting for scoring system. In the experiment with targets as the interval input, we extracted duplication/deletion regions covered by one or more consecutive probe-level significant duplications/deletions at the step of adding duplication or deletion region covered by consecutive strong probe-level CNV signals, and changed the threshold of "ProbeCntInRegion" filter to one or more and removed the threshold of "STDOfReadDepthRatios" filter to detect single-level exon CNVs.

Abbreviation	Description	Calculation method	Parameter setting for probe input interval	Parameter setting for target input interval
ProbeCntInRegion	How many signals support the CNV candidate?	Counting read depth ratio signals for a CNV candidate	1 point for ≥ 2	1 point for ≥ 1
AverageOfReadDepthRatios	How strong is the signal supporting the CNV candidate?	Calculating an average log2-transformed median predicted probe-level read depth ratio values for a CNV candidate	If deletion, 1 point for < $log_2(0.6)$; If duplication, 1 point for > log2(1.4)	If deletion, 1 point for < $log_2(0.6)$; If duplication, 1 point for > log2(1.4)
STDOfReadDepthRatios	How stable are the signals supporting the CNV candidate?	Calculating a standard deviation for the log2-transformed median predicted probe-level read depth ratio values for a CNV candidate	1 point for < 0.4	-
AverageOfCIs	How small are the confidence intervals for the signals supporting the CNV candidate?	Calculating average log2-transformed 95% confidence interval lengths for predicted probe-level read-depth ratios for a CNV candidate	1 point for < 0.4	1 point for < 0.4
AverageOfR2vals	How reliable is the model that generated the signals that support the CNV candidate?	Calculating average mean R-squared values per probe for a CNV candidate, with the average R-squared value per probe referring to an average of the R-squared values of N models for one probe	1 point for ≥0.85	1 point for ≥0.85

CNV, copy number variant; CI, confidence interval.

With total 14 cell-line known CNVs, we compared the performances of two experiments. In the PCR-based method using multiple pools, it was more useful to use amplicon information rather than target information as it detects fewer CNV candidates. This is thought to be due to low information loss. For the hybridization-based method, if probe information could not be obtained, the target information was used as the input interval. However, the probe interval input method with less information loss showed better performance when looking for small size CNVs (*GAA* EX18 DEL).

Sample			CNV				Probe interval input		Target interval input	
Panel	Cell line	Median read depth	Gene	NM	CNV	CNV size (kb)	Find?[a]	#CNV[b]	Find?	#CNV
IMD_HYB	GM14603	81.99	*GAA*	NM_000152	EX18 DEL	0.16	O	24	X	5
	GM14734	249.4	*CYP21A2*	NM_000500	30 KB DEL, Entire gene DEL	3.35	O	2	O	1

	GM24007	142.84	*OTC*	NM_000531	Entire gene DEL	68.97	O	7	O	7
	NA01741	164.4	*GALT*	NM_000155	Entire gene DEL	4.01	O	6	O	4
	NA06804	261.98	*HPRT1*	NM_000194	EX2-3 DUP	2.01	O	34	O	15
	NA06805	80.13	*GALC*	NM_000153	EX11-17 DEL	17.73	O	44	X	12
	NA12217	269.08	*CYP21A2*	NM_000500	30 KB DEL	1.14	X	1	O	3
	NA22208	199.64	*PCCA*	NM_000282	EX13-20 DEL	146.38	O	3	O	2
IMD_PCR	NA01741	Pool 1: 408.0, Pool 2: 556.0, Pool 3: 271.0	*GALT*	NM_000155	Entire gene DEL	4.01	O	10	O	5
	NA12217	Pool 1: 192.0, Pool 2: 117.0, Pool 3: 99.0	*CYP21A2*	NM_000500	30 KB DEL	1.14	X	37	X	14
	GM14603	Pool 1: 215.0, Pool 2: 141.0, Pool 3: 90.0	*GAA*	NM_000152	EX18 DEL	0.16	O	25	O	35
	NA14734	Pool 1: 359.0, Pool 2: 275.0, Pool 3: 335.0	*CYP21A2*	NM_000500	30 KB DEL, Entire gene DEL	3.35	O	9	O	123
	NA22208	Pool 1: 235.0, Pool 2: 99.0, Pool 3: 158.0	*PCCA*	NM_000282	EX13-20 DEL	146.38	O	27	O	148
	GM24007	Pool 1: 37.0, Pool 2: 20.0, Pool 3: 16.0	*OTC*	NM_000531	Entire gene DEL	68.97	X	1	X	0

CNV, copy number variation; IMD, inherited metabolism disorder; HYB, hybridization based capture approach; PCR, polymerase chain reaction based capture approach; EX, exon; DEL, deletion; DUP, duplication.

[a]Indicates whether each method found a known CNV. "O" means all CNVs were found, and "X" means they were not found at all; [b]indicates the number of CNV candidates found in the corresponding sample. The number of 5-score CNV candidates that received the highest score for the probe interval input method and the number of 4-score CNV candidates that received the highest score for the target interval input method is indicated.

Note S9. Performance comparison based on MQV thresholds

DeviCNV uses only reads that exceed the user-input mapping quality value (MQV) threshold. We compared cell-line CNV deletion results from DeviCNV using the following MQV thresholds: MQV≥0 and MQV≥20.

The statistics of raw CNV candidates, before scoring with CNV candidates by the score system, are shown in the following table. Overall, “MQV≥20” experiment using only reliable reads shows fewer raw CNV candidates.

Panel	#raw CNV	DeviCNV (MQV ≥ 0)				DeviCNV (MQV ≥ 20)			
		Failure rate[a]	DUP	DEL	TOTAL	Failure rate	DUP	DEL	TOTAL
IMD_HYB	Average	11% (7/66)	59.3	25.9	85.2	9% (9/66)	55.2	25.1	80.3
	Median		14	5	25		14	8	23
IMD_PCR	Average	3% (1/34)	47.9	71.3	119.2	3% (1/34)	48	70.7	118.7

	Median		35	28	76		33	31	68

Average, an average of raw CNV candidates of all samples except low-quality samples; Median, a median of all CNV candidates of all samples except low-quality samples; DEL, deletion; DUP, duplication; TOTAL, the total number of deletions and duplications.

[a]Indicates how many input samples fail in the low-quality sample filtering step.

The following table shows statistics for CNV candidates with confidence score of 5, which is the highest score from scoring system of DeviCNV.

Panel	#CNV	DeviCNV (MQV ≥ 0)				DeviCNV (MQV ≥ 20)			
		Failure rate[a]	DUP	DEL	TOTAL	Failure rate	DUP	DEL	TOTAL
IMD_HYB	Average	11% (7/66)	7.7	2.4	10.1	9% (9/66)	7.5	2.5	10.0
	Median		1	0	2		2	1	3
IMD_PCR	Average	3% (1/34)	14.4	8.2	22.6	3% (1/34)	14.1	7.9	22
	Median		6	4	10		6	3	11

Average, an average of CNV candidates with the highest score of 5 of all samples except low-quality samples; Median, a median of CNV candidates with the highest score of 5 of all samples except low-quality samples; DEL, deletion; DUP, duplication; TOTAL, the total number of deletions and duplications.

[a]Indicates how many input samples fail in the low-quality sample filtering step.

Comparison of the two experiments revealed that the number of the highest confidence CNV candidates and the performance of the known cell-line CNV detection were similar. The MQV threshold may affect the read depth calculation in authentic gene regions for genes with corresponding pseudogenes, since the reads in these regions had low MQV values.

In case of *GAA* single-exon deletion of GM14603 of IMD_HYB, the coverage of the corresponding sample was not good. Therefore, we confirmed that this CNV was only detected in MQV0 experiment, using as many reads as possible.

For the 1-copy deletion of *CYP21A2* of NA12217 samples of IMD_HYB/IMD_PCR, it was too difficult to detect due to the effect of pseudogene. We confirmed that the CNV was detected in the IMD_HYB MQV≥20 experiment using only reliable reads.

For the 1-copy deletion of *OTC* of GM24007 sample of IMD_PCR, the quality of this sample was bad for CNV calling, due to low coverage and filter-out from low-quality sample filter. Therefore, *OTC* deletion was not detected in all experiments.

Sample			CNV				DeviCNV (MQV ≥ 0)		DeviCNV (MQV ≥ 20)	
Panel	Cell line	Median read depth	Gene	NM	CNV	CNV size (kb)	Find?[a]	#CNV[b]	Find?	#CNV
IMD_HYB	GM14603	81.99	*GAA*	NM_000152	EX18 DEL	0.16	O	24	X	5

	GM14734	249.4	*CYP21A2*	NM_000500	30 KB DEL, Entire gene DEL	3.35	O	2	O	3
	GM24007	142.84	*OTC*	NM_000531	Entire gene DEL	68.97	O	7	O	11
	NA01741	164.4	*GALT*	NM_000155	Entire gene DEL	4.01	O	6	O	6
	NA06804	261.98	*HPRT1*	NM_000194	EX2-3 DUP	2.01	O	34	O	30
	NA06805	80.13	*GALC*	NM_000153	EX11-17 DEL	17.73	O	44	O	22
	NA12217	269.08	*CYP21A2*	NM_000500	30 KB DEL	1.14	X	1	O	2
	NA22208	199.64	*PCCA*	NM_000282	EX13-20 DEL	146.38	O	3	O	6
IMD_PCR	NA01741	Pool 1: 408.0, Pool 2: 556.0, Pool 3: 271.0	*GALT*	NM_000155	Entire gene DEL	4.01	O	10	O	11
	NA12217	Pool 1: 192.0, Pool 2: 117.0, Pool 3: 99.0	*CYP21A2*	NM_000500	30 KB DEL	1.14	X	37	X	35
	GM14603	Pool 1: 215.0, Pool 2: 141.0, Pool 3: 90.0	*GAA*	NM_000152	EX18 DEL	0.16	O	25	O	23
	NA14734	Pool 1: 359.0, Pool 2: 275.0, Pool 3: 335.0	*CYP21A2*	NM_000500	30 KB DEL, Entire gene DEL	3.35	O	9	O	9
	NA22208	Pool 1: 235.0, Pool 2: 99.0, Pool 3: 158.0	*PCCA*	NM_000282	EX13-20 DEL	146.38	O	27	O	31
	GM24007	Pool 1: 37.0, Pool 2: 20.0, Pool 3: 16.0	*OTC*	NM_000531	Entire gene DEL	68.97	X	1	X	2

CNV, copy number variation; IMD, inherited metabolism disorder; HYB, hybridization based capture approach; PCR, polymerase chain reaction based capture approach; MQV, mapping quality value; EX, exon; DEL, deletion; DUP, duplication.

[a]Indicates whether each method found a known CNV. "O" means all CNVs were found, and "X" means they were not found at all; [b]indicates the number of CNV candidates that received the highest score of 5 found in the corresponding sample.

Note S10. Low-quality sample filter by using sample-to-sample correlation

To filter out low-quality samples, we assumed that if the number of segments resulting from running a CBS (circular binary segmentation) of a sample is too many than other samples, the sample is considered as low-quality, which has high probability to detect abnormally large CNVs. Since large amount of CNV candidates from these low-quality samples are unreliable, they should be excluded when creating a linear regression model.

DeviCNV finds CNV candidates by comparing the read depth ratios of the samples in the same batch. Therefore, for samples with low sample-to-sample correlation values, the number of CNVs candidates must be high. Therefore, these samples must be filtered out in the generating model step.

To determine the criteria for the low-quality sample filter, we analyzed the histogram of CBS segments from the all samples of IMD_HYB and IMD_PCR. To count CBS segments

for each sample, only the number of segments with read depth ratio values of more than 1.3 or less than 0.7 (possibly a candidate for duplication/deletion) was selected.

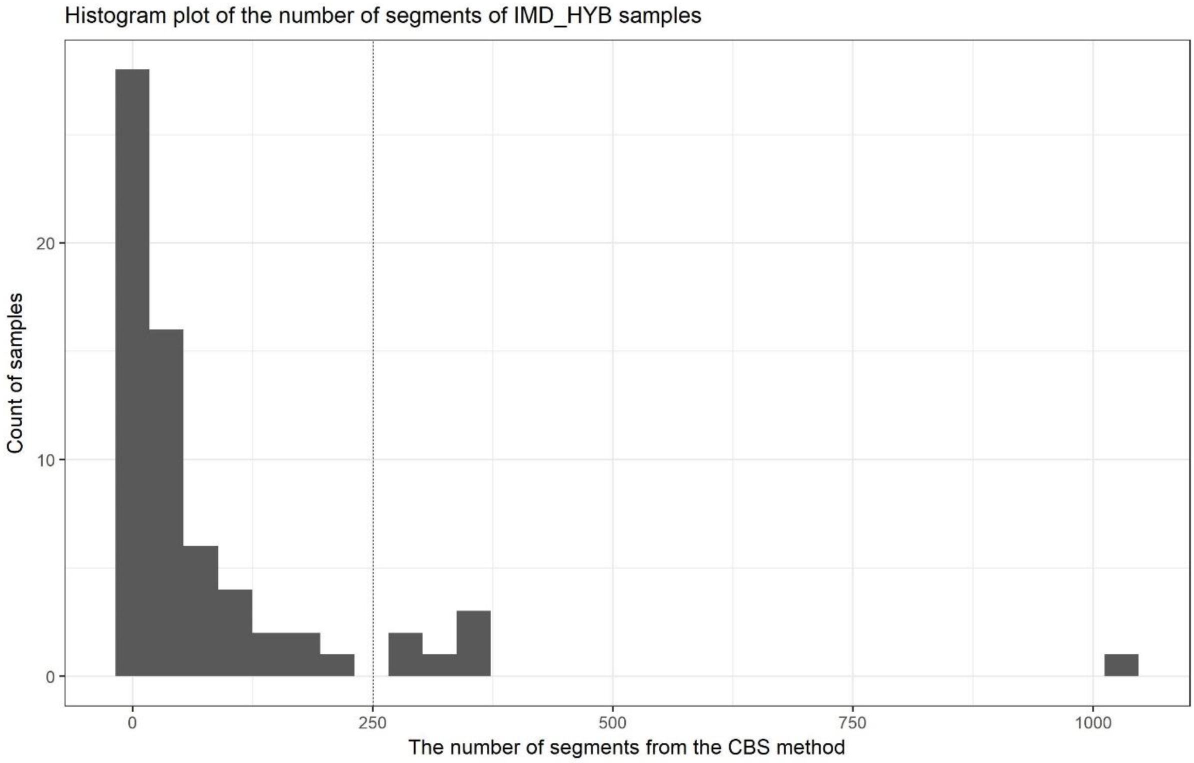

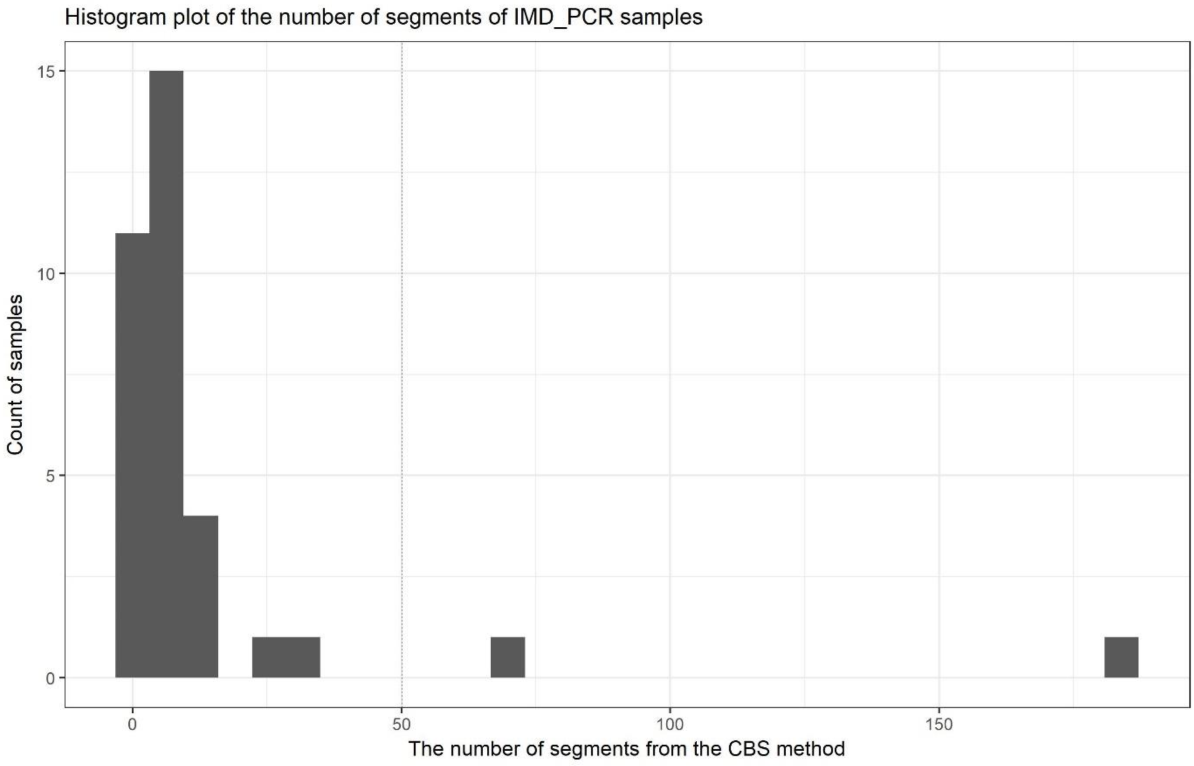

Samples with 250 or more segments in IMD_HYB and samples with 50 or more in IMD_PCR were defined as low-quality samples.

Then, we compared the number of segments of the samples, as well as the sample-to-sample correlation with other samples. In a batch of n samples, we compared one sample to the other samples (n-1) to obtain (n-1) correlation values for the 100th, 75th, 50th, 25th percentiles and the minimum, respectively. Each of these five sample-to-sample correlation values were compared with the number of segments in the sample, and the correlations between them were calculated. Finally, we selected the one with the highest correlation.

Sample-to-sample correlation	Correlation coefficient with the number of segments	
	IMD_HYB	IMD_PCR
The 100th percentile	−0.9209	−0.1646
The 75th percentile	−0.9363	−0.058
The 50th percentile	−0.9173	−0.0797
The 25th percentile	−0.8324	−0.0225
The minimum	−0.1135	−0.1936

IMD, inherited metabolism disorder; HYB, hybridization based capture approach; PCR, polymerase chain reaction based capture approach;

In the case of IMD_PCR analysis, the association between the number of segments and the sample-to-sample correlation value was low. For the IMD_HYB analysis, the 75th percentile value of the sample-to-sample correlation was -0.9363, which was most closely related to the number of segments.

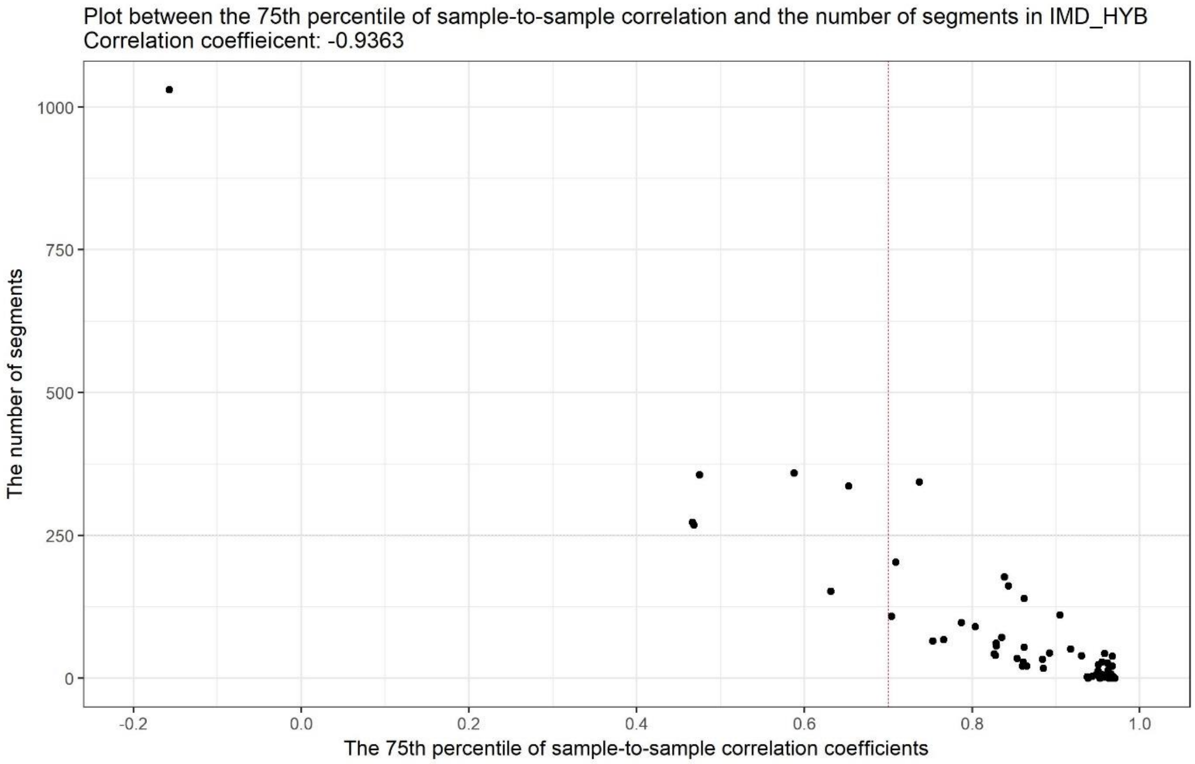

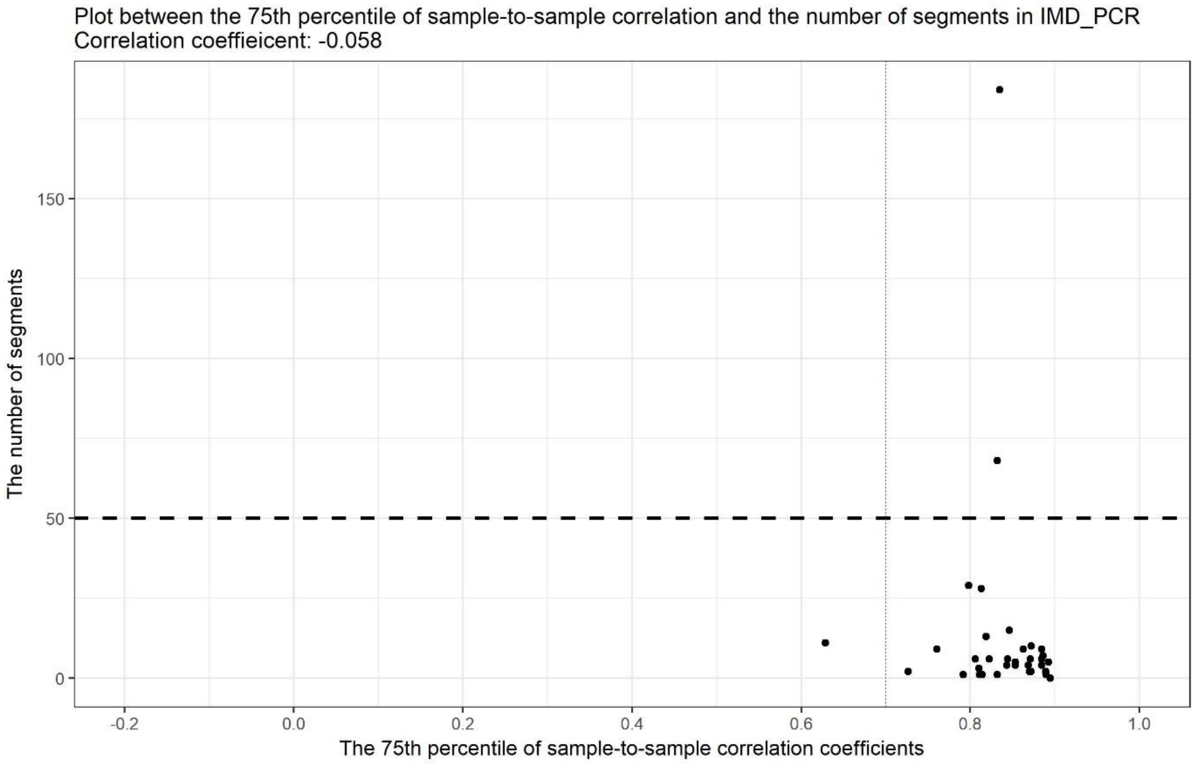

Finally, the 75th percentile sample-to-sample correlation value was considered to filter out the sample with a value less than 0.7.

Note S11. Performance comparison based on duplication and deletion thresholds for read depth ratios

When DeviCNV calculates p-values of probe-level CNVs and select raw CNV candidates, it needs duplication and deletion thresholds. We performed experiments with five duplication/deletion thresholds using IMD_HYB and IMD_PCR.

The following table shows statistics for raw CNV candidates before scoring. The stricter the duplication/deletion thresholds, the smaller the number of raw CNV candidates detected.

	TH.dup: 1.1 & TH,del: 0.9			TH.dup: 1.2 & TH.del: 0.8			TH.dup: 1.3& TH,del: 0.7			TH.dup: 1.4& TH,del: 0.6			TH.dup: 1.5& TH,del: 0.5		
#raw CNV	DUP	DEL	TOTAL	DUP	DEL	TOTAL	DUP	DEL	TOTAL	DUP	DEL	TOTAL	DUP	DEL	TOTAL
Average	427.7	410.8	838.5	153.6	112.6	266.1	59.3	25.9	85.2	24.7	6.9	31.6	11.8	2.4	14.2
Median	444	451	837	97	55	150	14	6	25	4	2	7	1	1	3

Average, an average of raw CNV candidates of all samples except low quality samples; Median, a median of raw CNV candidates of all samples except low quality samples; DEL, deletion; DUP, duplication; TOTAL, the total number of deletions and duplications.

The following table shows statistics for CNV candidates with confidence score of 5, which is the highest score from the scoring system of DeviCNV. DeviCNV extracts reliable CNV candidates through a scoring system. If the duplication/deletion thresholds are too low, it occurs a high probability that the pattern of data points in one CNV candidate is not stable, or the average read depth ratio value is not low(or high) enough. Therefore, it is understandable that 5-score CNV candidates are extracted less in the "TH.dup: 1.1 & TH, del: 0.9" experiment.

	TH.dup: 1.1 & TH,del: 0.9			TH.dup: 1.2 & TH.del: 0.8			TH.dup: 1.3& TH,del: 0.7			TH.dup: 1.4& TH,del: 0.6			TH.dup: 1.5& TH,del: 0.5		
# CNV	DUP	DEL	TOTAL	DUP	DEL	TOTAL	DUP	DEL	TOTAL	DUP	DEL	TOTAL	DUP	DEL	TOTAL
Average	4.9	1.5	6.4	5.6	1.7	7.3	8.8	2.4	11.2	10.9	3.2	14.1	3.8	1	4.8
Median	1	0	2	1	0	2	1	0	3	1	1	3	1	0	1

Average, an average of CNV candidates with the highest score of 5 of all samples except low quality samples; Median, a median of CNV candidates with the highest score of 5 of all samples except low quality samples; DEL, deletion; DUP, duplication; TOTAL, the total number of deletions and duplications.

Our cell-line and clinical data with known or confirmed CNVs were used to compare performance using various duplication/deletion thresholds. Results are shown in the table

below. When the duplication threshold was 1.3 and the deletion threshold was 0.7, all CNVs except two were found, and the performance was optimal. Therefore, we set these thresholds to 0.7 and 1.3, respectively, as default.

Sample			CNV				TH.dup: 1.1 & TH,del: 0.9		TH.dup: 1.2 & TH.del: 0.8		TH.dup: 1.3& TH,del: 0.7		TH.dup: 1.4& TH,del: 0.6		TH.dup: 1.5& TH,del: 0.5	
**Panel**	**Cell line**	**Median read depth**	**Gene**	**NM**	**CNV**	**CNV size (kb)**	Find?[a]	#CNV[b]	Find?	#CNV	Find?	#CNV	Find?	#CNV	Find?	#CNV
IMD_HYB	GM14603	81.99	*GAA*	NM_000152	EX18 DEL	0.16	O	8	O	15	O	24	O	27	X	8
	GM14734	249.4	*CYP21A2*	NM_000500	30 KB DEL, Entire gene DEL	3.35	O	2	A (EX1 copy-number error)	3	O	2	O	2	A (EX1-7)	1
	GM24007	142.84	*OTC*	NM_000531	Entire gene DEL	68.97	O	3	O	3	O	7	O	10	A (EX1-6,8-9)	6
	NA01741	164.4	*GALT*	NM_000155	Entire gene DEL	4.01	O	2	O	3	O	6	O	9	O	2
	NA06804	261.98	*HPRT1*	NM_000194	EX2-3 DUP	2.01	O	17	O	20	O	34	O	48	O	16
	NA06805	80.13	*GALC*	NM_000153	EX11-17 DEL	17.73	O	12	O	21	O	44	O	62	O	11
	NA12217	269.08	*CYP21A2*	NM_000500	30 KB DEL	1.14	X	1	A (EX1)	2	X	1	X	1	X	1
	NA22208	199.64	*PCCA*	NM_000282	EX13-20 DEL	146.38	A (larger region)	3	O	3	O	3	O	3	A (EX14, 18-20)	4
	Case_02	341.4	*ASL*	NM_000048	EX15 DEL	0.08	O	6	O	7	O	7	O	6	X	4
	Case_03	276.8	*GYS2*	NM_021957	EX6-11 DEL	5.15	O	5	O	5	O	5	O	7	A (EX8)	5
IMD_PCR	NA01741	Pool 1: 408.0, Pool 2: 556.0, Pool 3: 271.0	*GALT*	NM_000155	Entire gene DEL	4.01	O	13	O	14	O	10	O	8	O	2
	NA12217	Pool 1: 192.0, Pool 2: 117.0, Pool 3: 99.0	*CYP21A2*	NM_000500	30 KB DEL	1.14	X	46	X	55	X	37	X	15	X	4
	GM14603	Pool 1: 215.0, Pool 2: 141.0, Pool 3: 90.0	*GAA*	NM_000152	EX18 DEL	0.16	X	23	X	29	O	25	O	18	O	11
	NA14734	Pool 1: 359.0, Pool 2: 275.0, Pool 3: 335.0	*CYP21A2*	NM_000500	30 KB DEL, Entire gene DEL	3.35	O	10	O	12	O	9	O	5	O	3
	NA22208	Pool 1: 235.0, Pool 2: 99.0, Pool 3: 158.0	*PCCA*	NM_000282	EX13-20 DEL	146.38	O	11	O	19	O	27	O	11	A (EX14, 17)	5
	GM24007	Pool 1: 37.0, Pool 2: 20.0, Pool 3: 16.0	*OTC*	NM_000531	Entire gene DEL	68.97	X	0	X	0	X	1	X	0	X	2
	Case_04	Pool 1: 174.0 Pool 2: 203.0 Pool 3: 185.0	*ETFDH*	NM_004453	EX1-7 DEL	23.51	O	19	O	29	O	26	O	15	A (EX1-3, 5)	7
	Case_05	Pool 1: 228.0 Pool 2: 330.0 Pool 3: 185.0	*ETFDH*	NM_004453	EX7-8 DEL	2.20	X	54	X	72	O	63	X	37	X	13

CNV, copy number variation; IMD, inherited metabolism disorder; HYB, hybridization based capture approach; PCR, polymerase chain reaction based capture approach; TH.dup, duplication threshold for read depth ratio; TH.del, deletion threshold for read depth ratio; EX, exon; DEL, deletion; DUP, duplication.

[a]Indicates whether each method found a known CNV. "O" means all CNVs were found, "X" means they were not found at all, and "A" means they were found only in some exons; [b]indicates the number of CNV candidates that received the highest score of 5 found in the corresponding sample.

Note S12. Unique scoring system for selecting high-confidence CNV candidates

To achieve single exon resolution CNV detection, DeviCNV selects even small size CNVs, and that leads to many CNV candidates that need to be validated by the user. Moreover, if the input data are of low quality, many CNV candidates are detected because of the noise. Therefore, we have implemented a unique priority calculation system for raw CNV candidates in DeviCNV, to provide users with highly reliable CNV candidates. We selected five statistical results as measures of CNV confidence. The descriptions and calculation methods for the five measures are described below. After setting the optimal threshold for the above five measures, DeviCNV counts the number of measures that satisfy the criteria (the weights for all measures are 1). This allows candidates with high scores to be the highest priority candidates, thereby reducing the number of candidates the user must identify and verify. If all the measures are satisfied, the corresponding CNV candidate has a maximum value of five points.

(1) *ProbeCntInRegion*: how many signals support the CNV candidate
We count the number of probe-level read depth ratios for the CNV candidate. The larger this value is, the higher the reliability of the CNV candidate, because this indicates that many signals support that candidate. If the user only wants to get CNV candidates supported by more multiple probes, user can increase this value.

(2) *AverageOfReadDepthRatios*: how strong the signal supporting the CNV candidate
This value represents how far an average of the median read depth ratios supporting the CNV candidate is from the normal state (read depth ratio = 1). In the gene-centric plot, the red rectangle indicates the median read depth ratio, and the average of these values is *AverageOfReadDepthRatios*. The smaller this value is, the higher the reliability of detecting a deletion candidate. In contrast, the larger this value is, the higher the reliability of detecting a duplication candidate.

(3) *STDOfReadDepthRatios*: how stable the signals supporting the CNV candidate
It indicates whether the read depth ratios supporting the corresponding CNV candidates are constant. Thus, even if the median read depth ratios are far from 1(=neutral copy number) for a given CNV candidate, if the standard deviation of these values is large, this does not represent a stable signal. Therefore, the standard deviation of the median read depth ratios is obtained as *STDOfReadDepthRatios*. The closer this value is to zero, the more reliable the CNV candidate is.

(4) *AverageOfCIs*: how small the confidence intervals of the signals supporting the CNV candidate

In the gene-centric plot, the whisker of each red box (read depth ratio) represents the 95% confidence interval of the signal. The average of the confidence interval length of read depth ratios supporting the CNV candidate is *AverageOfCIs*. The closer this value is to zero, the greater the reliability of the CNV candidate.

(5) *AverageOfR2vals*: how reliable the model that generated the signals that support the CNV candidate

The R-squared values of the models indicate how the data fits the regression line. For example, the predicted read depth ratios from a model with a low R-squared value are not well predicted from the regression, and so they are more likely to represent noise and thus be less reliable. After calculating an average of the R-squared values of the total N models used to calculate one read depth ratio signal, an average of the average R-squared values for the signals supporting one CNV candidate is considered as *AverageOfR2vals*. The closer this value is to 1, the greater the reliability of the CNV candidate.

Note S13. Inherited metabolic disorder (IMD) panel description

We have developed a custom gene panel for targeted DNA sequencing for clinical diagnosis and newborn screening covering inborn errors of metabolisms, and other diseases. The current version of the inherited metabolic disorder (IMD) panel is composed of 259 target genes and covers 498,034 bps of target regions, and there are two sub-panels for hybridization and polymerase chain reaction (PCR)-based capture approaches: IMD_HYB and IMD_PCR, respectively. The previous version of the IMD panel (IMD_V1) contained 97 genes, covering 150,802 bps. This is the only version including a PCR-based approach.

Note S14. Generating targeted NGS data

Targeted NGS data from cell lines and clinical samples were generated for validation and identification of known cell-line copy number variants (CNVs) and pathogenic CNVs.

To briefly describe the hybridization-based capture approach, sample DNA was sheared to around 150 bp, and barcoded adapters were ligated to allow the DNA fragments to be captured and sequenced. The fragments belonging to the target region were captured, purified, and sequenced using the 100 bp paired-end setting on a HiSeq instrument. Sequenced reads were aligned using the BWA version 0.7.12[2], and duplicates were marked and sorted using Picard 1.139 (http://broadinstitute.github.io/picard/). The reads were also realigned and recalibrated using the Genome Analysis Toolkit version 3.4.46 (GATK)[3, 4].

For the PCR-based capture approach, locus specific primers with barcoded adapters were designed to attach to a targeted region during PCR. Following PCR amplification, the amplified DNA fragments were pooled, purified, and sequenced on an Ion Torrent Personal Genome Machine (PGM) system or S5 (Thermo Fisher Scientific, Waltham, MA, USA). The data were processed for alignment with the standard Ion Torrent Suite™ Software on the Torrent Server.

Note S15. Failure rate of DeviCNV, VisCap, XHMM, and CODEX

The performance of DeviCNV was compared to VisCap, XHMM, and CODEX. The following table shows failure rates after sample QC for each tool.

In the case of VisCap, there is 'run_1' which calls CNV using all samples. After that, 'run_2', which calls CNV again, is repeated while removing samples with low quality. This process is performed until all remaining samples pass their own low-quality sample filter, and the number of runs per batch is different. The following table shows failure rates for 'run_1', 'run_2', and 'run_last', the last run of the batch. For most batches in all panels of VisCap, the failure rates were high when 'run_2'. Therefore, the result of 'run_1' was used for comparison with DeviCNV.

Tool		Panels	IMD_HYB		IMD_PCR		IMD_V1
		Batches	3		2		Unknown
		Samples	30 (cell line)	36 (clinical)	14 (cell line)	20 (clinical)	178 (clinical)
		Average depth of coverage	174X	345X	301X	349X	87X
DeviCNV		Samples passing QC	24	35	14	19	172
		Failure rate	20%	3%	0%	5%	3%
VisCap	run_1	Samples passing QC	30	36	14	20	178
		Failure rate	0%	0%	0%	0%	0%

	run_2	Samples passing QC	1	15	0	0	174
		Failure rate	97%	58%	100%	100%	2%
	run_last	Samples passing QC	0	13	-	-	30
		Failure rate	100%	56%	-	-	83%
XHMM		Samples passing QC	25	35	10	17	166
		Failure rate	16.67%	2.78%	29%	15%	6.74%
CODEX		Samples passing QC	N/A	N/A	N/A	N/A	N/A
		Failure rate	N/A	N/A	N/A	N/A	N/A

CNV, copy number variation; QC, quality control; IMD, inherited metabolism disorder; HYB, hybridization based capture approach; PCR, polymerase chain reaction based capture approach.

Note S16. List of abbreviations

CNV: Copy number variant; NGS: Next generation sequencing; SNV: Single-nucleotide variant; INDEL: Short insertion and deletion; IMD: Inherited Metabolism Disorders; PCR: polymerase chain reaction; qPCR: Quantitative polymerase chain reaction; PGM: The Ion Torrent Personal Genome Machine; GATK: Genome Analysis Toolkit; QC: Quality control; BAM: Binary Alignment/Map; MQV: Mapping quality value; PCA: Principal component analysis; CBS: Circular binary segmentation; Bp: Base pairs; CN: Copy number; DUP: Duplication; DEL: Deletion; NEU: Neutral; CI: Confidence interval.

References

1. Li H, Handsaker B, Wysoker A, Fennell T, Ruan J, Homer N, Marth G, Abecasis G, Durbin R, Genome Project Data Processing S: **The Sequence Alignment/Map format and SAMtools**. *Bioinformatics* 2009, **25**(16):2078-2079.
2. Li H: **Aligning sequence reads, clone sequences and assembly contigs with BWA-MEM**, vol. 1303; 2013.
3. DePristo MA, Banks E, Poplin R, Garimella KV, Maguire JR, Hartl C, Philippakis AA, del Angel G, Rivas MA, Hanna M *et al*: **A framework for variation discovery and genotyping using next-generation DNA sequencing data**. *Nature Genetics* 2011, **43**:491.
4. McKenna A, Hanna M, Banks E, Sivachenko A, Cibulskis K, Kernytsky A, Garimella K, Altshuler D, Gabriel S, Daly M *et al*: **The Genome Analysis Toolkit: A MapReduce framework for analyzing next-generation DNA sequencing data**. *Genome Research* 2010, **20**(9):1297-1303.
